# Reprogramming the immunosuppressive tumor microenvironment: exploiting angiogenesis and thrombosis to enhance immunotherapy

**DOI:** 10.3389/fimmu.2023.1200941

**Published:** 2023-07-03

**Authors:** Areez Shafqat, Mohamed H. Omer, Eman Nayaz Ahmed, Ali Mushtaq, Eman Ijaz, Zara Ahmed, Khaled Alkattan, Ahmed Yaqinuddin

**Affiliations:** ^1^ College of Medicine, Alfaisal University, Riyadh, Saudi Arabia; ^2^ School of Medicine, Cardiff University, Cardiff, United Kingdom; ^3^ Department of Internal Medicine, Cleveland Clinic Foundation, Cleveland, OH, United States

**Keywords:** immunotherapy, tumor microenvironment, angiogenesis, thrombosis, vascular normalization, hypoxia, treatment resistance

## Abstract

This review focuses on the immunosuppressive effects of tumor angiogenesis and coagulation on the tumor microenvironment (TME). We summarize previous research efforts leveraging these observations and targeting these processes to enhance immunotherapy outcomes. Clinical trials have documented improved outcomes when combining anti-angiogenic agents and immunotherapy. However, their overall survival benefit over conventional therapy remains limited and certain tumors exhibit poor response to anti-angiogenic therapy. Additionally, whilst preclinical studies have shown several components of the tumor coagulome to curb effective anti-tumor immune responses, the clinical studies reporting combinations of anticoagulants with immunotherapies have demonstrated variable treatment outcomes. By reviewing the current state of the literature on this topic, we address the key questions and future directions in the field, the answers of which are crucial for developing effective strategies to reprogram the TME in order to further the field of cancer immunotherapy.

## 1 Introduction

Immunotherapies have revolutionized cancer treatment; however, their efficacy remains limited to a certain select tumor types primarily due to tumor immune evasion mechanisms. A key pathway through which tumors evade the immune system is the reprogramming of cellular constituents of the tumor microenvironment (TME) towards an immunosuppressive phenotype. Thus, enhancing the effectiveness of immunotherapies by manipulating the TME is a major focus of current research.

The tumor vasculature is key in controlling immune cell infiltration into tumors. However, tumor blood vessels can be highly abnormal, characterized by tortuous, primitive, and leaky vessels with an erratic blood flow, impeding effective immune cell trafficking into tumors. Such an abnormal vasculature results in areas of the tumor not receiving adequate oxygen, leading to tumor hypoxia that has separate downstream immunosuppressive effects. From a therapeutic standpoint, anti-angiogenic drugs have been in the market for nearly 20 years and have been shown to enhance tumor blood flow by normalizing the tumor vasculature and mitigating the downstream immunosuppressive effects of neoplastic angiogenesis. In recent years, several clinical trials have evaluated the efficacy of combining anti-angiogenic agents with immune checkpoint inhibitors (ICIs), and these combination regimens are now approved for the treatment of lethal cancers such as renal cell carcinoma (RCC) and hepatocellular carcinoma (HCC).

Tumor coagulation, broadly known as cancer-associated thrombosis (CAT), manifested in the form of venous thromboembolisms (VTEs) is a frequent complication in cancer patients. Components within the TME involved in hemostasis, collectively termed the tumor coagulome, have recently been shown to reshape the TME, thereby modulating immunotherapy response. These findings have paved the way for studies aiming to enhance immunotherapy responses by administering concomitant anticoagulation.

In this review, we aim to provide a comprehensive analysis of these processes, shedding light on their roles in fostering an immunosuppressive TME and current challenges regarding their potential as therapeutic targets in clinical settings.

## 2 Tumor angiogenesis

### 2.1 The angiogenic shift in tumors

Tumors initially exist in an avascular stage (i.e., without blood vessels), which limits their growth and metastatic potential. The “angiogenic shift” is pivotal for tumor survival, marking their transition from an avascular state to a vascularized one. This shift involves significant adaptations in the TME to create a pro-angiogenic environment (1).

One prominent metabolic alteration observed in cancer cells is the upregulation of glycolysis even under well-oxygenated conditions, known as “aerobic glycolysis” or the “Warburg effect” (2). Hypoxia-inducible factors (HIFs), which are transcription factors activated in response to low oxygen levels, play a central role in this metabolic rewiring of cancer cells by inducing a state of “pseudohypoxia”, redirecting cellular metabolism towards glycolysis (3, 4). This metabolic shift leads to the accumulation of lactate and tumor acidosis (5), exacerbated by poor tumor perfusion and the high metabolic demands of rapidly dividing cancer cells, which promote hypoxia and anaerobic glycolysis (6). In the hypoxic and acidotic TME, HIF-mediated gene expression changes enable tumor cells to survive in an otherwise inhospitable milieu (7).

HIFs are heterodimeric, composed of an alpha subunit (HIF-α) and a beta-subunit (HIF-β) (8). Activation of HIF-1α triggers the upregulation of pro-angiogenic mediators, including vascular endothelial growth factors (VEGF), platelet-derived growth factors (PDGF), and fibroblast growth factors (FGF) (9). Among these, VEGF plays a particularly crucial role in tumor angiogenesis (10–12).

### 2.2 Dysfunctional vessels in neoplastic angiogenesis

However, tumor angiogenesis often results in the formation of tortuous and leaky blood vessels with an erratic blood flow, leading to regions with poor blood flow and inadequate oxygenation (13). Tumor endothelial cells harbor numerous cytogenetic abnormalities, rendering them molecularly and morphologically unstable (14). VEGF signaling disrupts gap junctions between endothelial cells, increasing vascular permeability and interstitial hydrostatic pressure (15, 16). Furthermore, the detachment of pericytes from endothelial cells promotes vessel fragility and intra-tumoral hemorrhage (17–19). Additionally, the proteolytic degradation of the vascular basement membrane facilitates tumor cell intravasation and metastasis (20).

## 3 Angiogenesis reprograms the tumor microenvironment towards immunosuppression

In this section, we will delve into the multifaceted immunosuppressive effects of neoplastic angiogenesis on the TME, focusing on the direct effects of VEGF, tumor hypoxia, and acidosis.

### 3.1 Direct effects of angiogenic factors

VEGF downregulates the expression of leukocyte adhesion molecules such as ICAM-1 and VCAM-1 on endothelial cells, thereby inhibiting the infiltration of CD8^+^ T-cells into tumors (21–23). Strategies aimed at vascular normalization, such as anti-VEGF medications or p21-activated kinase-4 (PAK4) inhibition, can restore the expression of adhesion molecules and enhance CD8^+^ T-cell infiltration (24, 25). Endothelial cells express PD-L1 and Fas ligand, which suppress CD8^+^ T-cell effector functions and promote T_regs_-mediated immunosuppression (26, 27). Anlotinib, a VEGF receptor blocker, has been shown to downregulate endothelial PD-L1 and increase the ratio of CD8^+^ T-cell infiltration to T_regs_ (27). Additionally, VEGF promotes T-cell exhaustion, impairs dendritic cell (DC) maturation and function, recruits immunosuppressive cells like VEGR^+^ T_regs_, MDSCs, and pro-tumor M2 tumor-associated macrophages (TAMs), and contributes to an hypoxic and acidotic environment through the generation of dysfunctional vasculature (28–30).

### 3.2 Tumor hypoxia

**Figure 1:**
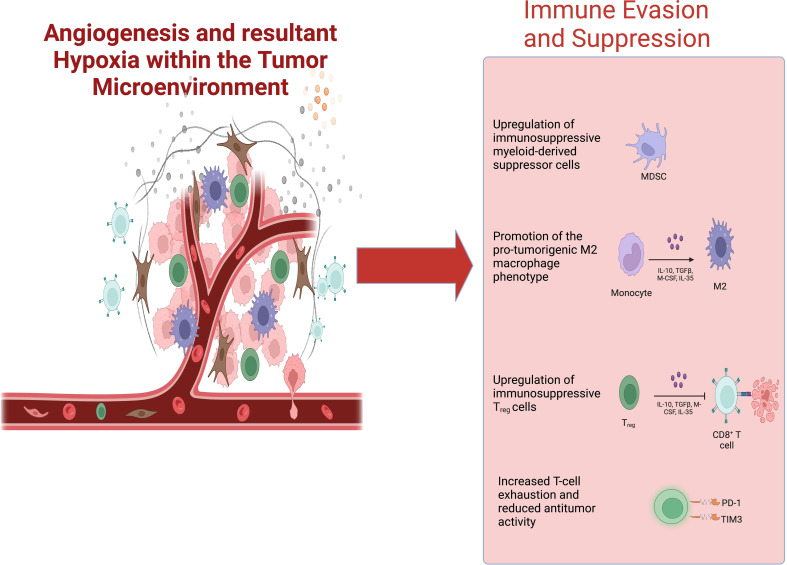
Pro-angiogenic signaling within the TME fosters tumor hypoxia. Tumor hypoxia generates an immunosuppressive tumor microenvironment— promoting the infiltration of MDSCs, M2 TAMs, T_regs_, and exhausted CD8^+^ T-cells—that negatively impacts the efficacy of cancer immunotherapies. Abbreviations: TME, tumor microenvironment; MDSCs, myeloid-derived suppressor cells; TAMs, tumor-associated macrophages.

Hypoxia skews cells of the TME towards immunosuppressive phenotypes (**Figure 1**). Hypoxic regions within tumors serve as niches where immunosuppressive cells, such as myeloid-derived suppressor cells (MDSCs), M2 TAMs, exhausted CD8^+^ T-cells, and T_regs_, preferentially accumulate (31). Pharmacologically inhibiting HIF-1/2 by 32-134D has been shown to downregulate genes involved in angiogenesis, glycolysis, and immune evasion. It also decreases the number of pro-tumorigenic M2 TAMs and MDSCs while increasing the infiltration of anti-tumor CD8^+^ cytotoxic T-cells and NK cells (32). Combining 32-134D with anti-PD-1 immune checkpoint inhibitors (ICIs) enhances therapy response (32). Additionally, inhibiting HIF-1α with echinomycin decreases PD-L1 expression on tumor cells, TAMs, and MDSCs when combined with anti-CTLA4 ICI therapy (33).Importantly, echinomycin augmented PD-L1 expression in normal tissues, promoting tolerance and protecting against immune-related adverse effects of ICIs (33).

TAMs play crucial roles in tumor growth, survival, and therapy resistance, which are directly correlated with HIF-1α- expression in these cells (34–36). Hypoxia upregulates the triggering receptor on myeloid cells-1 (TREM-1) receptor on TAMs, which skews naïve T-cells towards T_regs_ (37). Additionally, hypoxia induces an immunosuppressive M2 phenotype in TAMs by upregulating HIF-2α (38–40). If HIF-1α and HIF-2α are upregulated simultaneously in TAMs, whether one isoform predominates over the other, and the temporal dynamics based on type of hypoxia (intermittent vs continuous) or tumor stage remain unclear (41).

Tumor-associated neutrophils (TANs) have both pro-tumor and anti-tumor roles in cancer (42). Hypoxia prolongs neutrophil lifespan, enhances their degranulation function (43, 44), but attenuates the respiratory burst (45). Tumor hypoxia induces TAN recruitment through IL-8 and skews their phenotypes towards PMN-MDSCs, which suppress anti-tumor T-cell responses and promote tumor proliferation through neutrophil elastase (NE) (46–48). Reversing this phenotype by hyperoxia enhances anti-tumor immunity and tumor cell apoptosis (48).

VEGF impairs the differentiation of immature DCs into effective antigen-presenting mature DCs (49–51). Hypoxic regions of hepatocellular carcinoma harbor type-2 conventional dendritic cells (cDC2s) and immunosuppressive plasmacytoid dendritic cells, linked to the T_regs_ accumulation and CD8^+^ T-cell suppression (31, 52). T_regs_, in turn, can downregulate surface HLA-DR expression on cDC2s, impairing their antigen-presenting function (53). Contrarily, hypoxia activates various anti-tumor functions in DCs such as pro-inflammatory cytokine secretion (54). cDC2s have also been shown to modulate tumor evasion from CD8^+^ T-cell cytotoxicity (55). Such data indicate that all-or-none approaches targeting dendritic cells may be unsuccessful and/or exert unwanted pro-tumorigenic side effects, highlighting the need for elucidating the extrinsic (environmental) and intrinsic regulators of DC plasticity (56).

The upregulation of HIF-1α in naïve T-cells favors T_reg_ differentiation (57) and indirectly augments T_reg_ recruitment through CCL28 and TGF-β production in the TME (58, 59). Persistent hypoxia and antigen stimulation drive CD8^+^ T-cell exhaustion (60), with HIF-1α driving AMP production, which contributes to T-cell suppression and therapy resistance (60–62). However, conflicting data that observe HIF-1α and HIF-2α also support CD8^+^ T-cell proliferation and anti-tumor activity (36, 63, 64). Different types of tumor hypoxia (continuous vs. intermittent) may have varying effects on HIF-1α and HIF-2α (65, 66). Future research is required to elucidate the major hypoxia-related signaling pathways driving T-cells into anti-tumor or immunosuppressive phenotypes, along with their associated cell surface receptors and extrinsic regulators in the TME.

Cancer-associated fibroblasts (CAFs) play diverse roles in tumor progression (see (67) for detailed review). Increased CAFs presence in esophageal cancer correlates with decreased CD8^+^ cytotoxic T-cells and increased T_regs_ infiltration (68). CAFs secrete IL-6, which activates HIF-1α in tumor cells, augmenting their glucose uptake and glycolysis (69), which may stabilize T_regs._ IL-6 also induces the differentiation of fibroblasts into CAFs and TAMs to adopt an M2 polarization (69). An anti-IL-6 antibody slows tumor growth by increasing CD8^+^ T-cell infiltration and decreasing T_regs_ presence (68).

### 3.3 Tumor *acidosis*

Mechanistically, tumor acidosis induces an M2 phenotype on TAMs (70, 71), which release HMGB1 and arginase-1 that activate signaling pathways enhancing aggressive cancer phenotypes (72, 73). Tumor-derived lactic acid suppresses antigen-presenting functions of DCs to blunt T-cell activation (74), as well as directly attenuating CD8^+^ T and NK cell effector functions (75–79). Increasing extracellular pH by administering bicarbonate slows tumor growth and increases the infiltration of anti-tumor CD8^+^ T-cell s (80). Additionally, combining bicarbonate with anti-CTLA4 or anti-PD-L1 ICI improves therapy response (80).

The rapid consumption of glucose by tumor cells due to the Warburg effect limits glucose availability for CD4^+^ and CD8^+^ effector T-cells, favoring their suppression (81–87). This low glucose environment favors the functional stabilization of T_regs_ (88–91), as they can preferentially utilize fatty acids and lactate as metabolic substrates (92, 93). T_regs_ largely avoid glycolysis because high glucose concentrations in the TME and cellular uptake impair T_reg_ function (93). The T_reg_ avoidance of glucose is controlled by surface CTLA-4 and PD-1 (94, 95). However, chronic exposure to lactate, when studied apart from its acidic TME, increases the stemness of CD8^+^ T-cells and augments anti-tumor CD8^+^ immunity to suppress tumor growth (96, 97). Therefore, current research indicates that there exists a combinatorial influence of TCR signaling, hypoxia, low glucose, tumor acidosis, and lactate on the T-cell phenotype in the TME, with their combined effects potentially overshadowing lactate’s anti-tumor effects.

## 4 The tumor coagulome and thrombosis

Venous thromboembolic events (VTEs) are a common complication in several cancer types and remain a leading cause mortality in these patients (98–100). This discussion focuses on key aspects of the tumor coagulome as a mediator of CAT.

### 4.1 Coagulation *cascade*

The extrinsic coagulation pathway is initiation by tissue factor (TF), a transmembrane protein usually expressed by perivascular cells that is normally shielded from circulation, only being exposed after blood vessel damage. Once exposed, TF binds and activates factor VII, leading to the cleavage and activation of factor X into factor Xa (fXa), ultimately generating thrombin.

TF is the most extensively studied pro-coagulant in cancer-associated VTEs (101–105). TF levels are elevated in cancer patients due to TF upregulation on the surface of cancer cells, promoting extravascular thrombosis. Tumor cells and non-tumor immune cells in the TME also secrete extracellular vesicles (EVs) expressing TF (TF^+^-EVs), promoting intravascular thrombosis (106). TF-expressing tumor cells can also enter the circulation and induce CAT (107). TF upregulation in cancer is influenced by multiple factors, including genetic mutations, growth factors, inflammatory cytokines, and hypoxia (108). Beyond thrombosis, TF promotes cancer cell survival, proliferation, invasion, and metastasis (108). A recent study developed TF-chimeric antigen receptor natural killer (NK) cells that effectively target TF-overexpressing triple-negative breast cancer cells to decrease tumor growth without significant systemic adverse effects (109).

### 4.2 Platelets

Many cancer patients also display elevated serum levels of platelet-derived EVs and p-selectin in their serum, indicating systemic platelet activation. Tumor cell-induced platelet activation (TCIPA) involves multiple mechanisms (110–112). For example, the glycoprotein podoplanin (PDPN) expressed on the surface of many tumor cells binds the C-type lectin receptor-2 (CLEC-2) on the platelets leading to platelet aggregation and thrombus formation (113–115). Similarly, TF can directly activate platelets or facilitate tumor cell-platelet interactions (116, 117). EVs released by triple-negative breast cancer cells contain uPAR and PDGFRβ that can induce platelet aggregation (118). Platelet activation leads to degranulation and release of ADP and thromboxane A2, which can function in an autocrine manner to amplify platelet activation and aggregation (119). Other than CAT, platelets promote various other aspects of tumor progression, including sustained proliferative signaling, angiogenesis, epithelial-to-mesenchymal transition, immune evasion, and metastasis (120, 121).

### 4.3 Neutrophil extracellular traps

Neutrophils are known to produce neutrophil extracellular traps (NETs), which have been implicated in various pro-thrombotic diseases, including CAT (122). In cancer patients, NET markers such as extracellular DNA, myeloperoxidase (MPO), citrullinated histones, and NE are elevated and correlate positively with the incidence of VTEs (123–126). Moreover, NET components like DNA and cit-H3 are richly found in cancer-associated thrombi in mice and humans (125, 127–131). Circulating neutrophils retrieved from the blood of tumor-bearing mice or cancer patients are more prone to form NETs ex vivo (132).

Cancer cells create a systemic environment that promotes NETosis (132). Factors such as GM-CSF, IL-1β, CXCR1/CXCR2 agonists, cathepsin C, complement 5a, and EVs have been shown to promote NET formation in animal tumor models (133–139). Alternatively, tumors can stimulate NETosis through TCIPA, as activated platelets directly interact with neutrophils *via* p-selectin and high-mobility group box-1 (HMGB1), leading to NET production (140–142). Studies have shown that exogenous administration of Dnase-1, an enzyme that degrades NETs, or genetic deletion of peptidyl arginine deaminase-4 (PAD4) a protein essential for NETosis, significantly reduce thrombotic events and organ damage in mouse models of cancer (127, 128, 143). However, it is important to consider the risk of adverse events, such as infections, when using Dnase-1 therapy in immunosuppressed cancer patients (144).

## 5 The coagulome reprograms the tumor microenvironment towards immunosuppression

**Figure 2:**
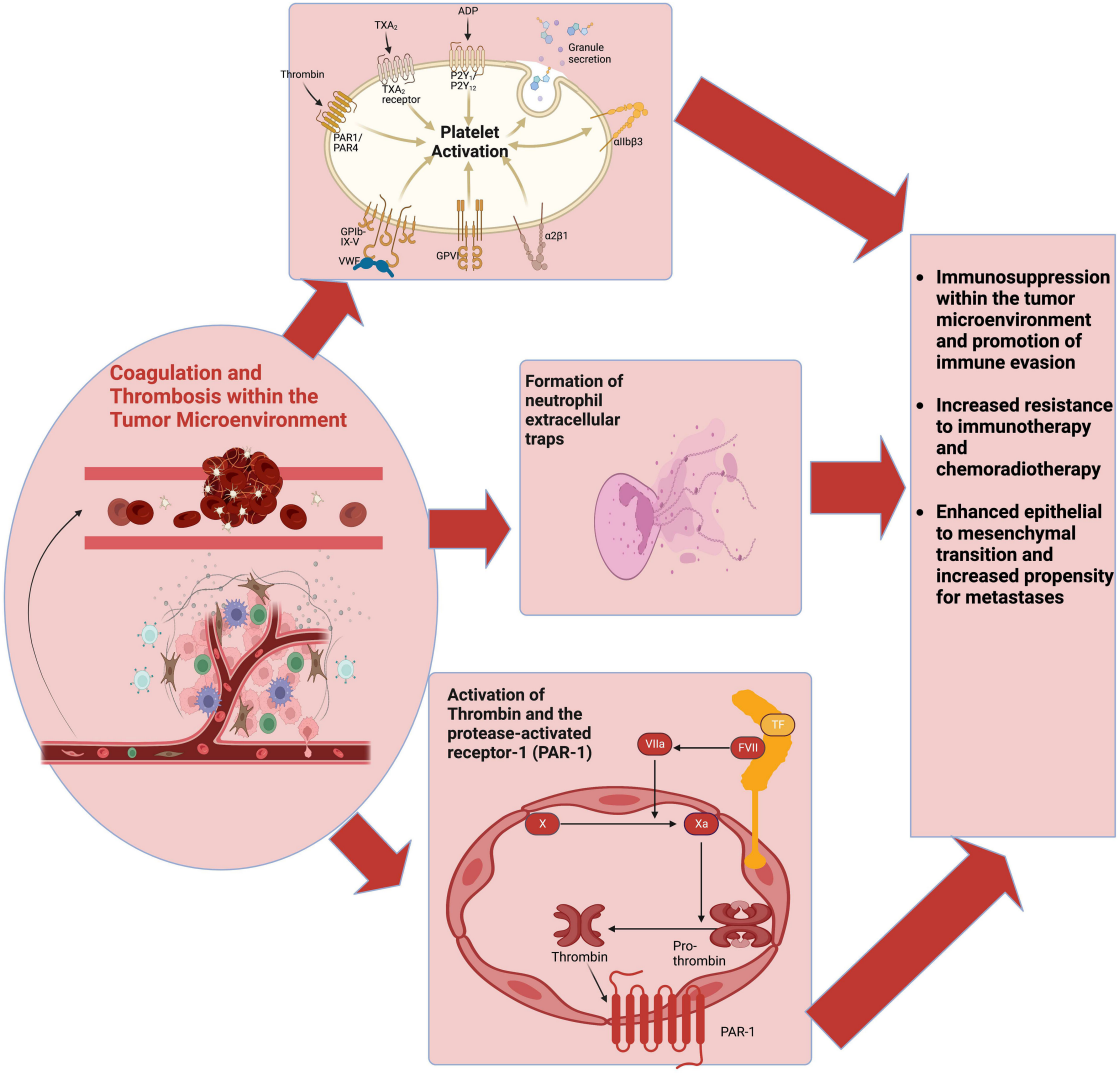
The coagulome describes various components of the tumor microenvironment that modulate coagulation. CAT involves the activation of multiple pathways, including platelet activation, the coagulation cascade, and NETs production. These pathways also interact with various cells of the tumor immune microenvironment to facilitate tumor immune evasion and immunotherapy resistance. Abbreviations: CAT, cancer-associated thrombosis; NETs, neutrophil extracellular traps.

Preclinical studies have demonstrated that the tumor coagulome fosters an immunosuppressive TME (**Figure 2**).

### 5.1 Coagulation cascade

Within the TME, TAM-derived fXa promotes the expansion of MDSCs while inhibiting CD8^+^ and NK cell functions, resulting in immunosuppression (145). Genetic depletion or pharmacologic inhibition of fXa using rivaroxaban enhances the anti-tumor response and improves the efficacy of ICIs in murine models of colorectal cancer and melanoma (145). Similarly, high levels of plasminogen activator inhibitor-1 (PAI-1) in murine lung carcinoma models increases increase the recruitment of TAMs and polarization towards an M2 phenotype whereas reducing PAI-1 levels decreases M2 TAMs and increases M1 TAMs (146).

Thrombin and protease-activated receptor 1(PAR1) signaling has been shown to promote tumor growth, metastasis, and immune evasion in murine pancreatic adenocarcinoma (PDAC) models (147). Mechanistically, PAR1 signaling in PDAC cells downregulates their antigen-processing machinery, resulting in these cells being less efficiently recognized by anti-tumor immune responses, and upregulates immunoregulatory proteins like GM-CSF, involved in recruiting immunosuppressive M2 TAMs and MDSCs. Furthermore, PAR1-dependent tumor growth is mediated through the upregulation of cyclooxygenase-2 (COX-2) and GM-CSF, indicating that these mediators could be potential targets for therapies inhibiting PDAC growth (148).

However, thrombin has also been shown to induce effector T-cell activation, proliferation, and cytokine production in various non-neoplastic contexts (149–151). Furthermore, several mediators of the coagulation cascade besides thrombin also activate PARs, indicating that the outcomes of PAR signaling is multifactorial, depending on the specific ligands, PAR subtypes, and tumor context (152). For example, thrombin-PAR1 signaling does not promote tumor progression in transgenic adenocarcinoma mouse prostate (TRAMP) models, while protein C binding to PAR1 promotes tumor cell apoptosis and slows the progression of prostate and intestinal cancers (153). Based on these discrepancies, we advocate for caution when considering long-term thrombin or PAR1 inhibition as an anti-cancer strategy.

A recent study found that reducing prothrombin levels in mice before treatment with ICIs led to decreased CD8^+^ T-cell infiltration and compromised anti-tumor immunity, resulting in a complete loss of therapeutic efficacy (154). Inoculating human CD8^+^ T-cells with thrombin increased their activation, even cells lacking PAR1 and PAR2, indicating the presence of independent mechanisms for thrombin-induced CD8^+^ T-cell activation. However, the study also revealed that PAR2 signaling suppresses T-cell activation and attenuates thrombin/PAR1-dependent activation of anti-tumor CD8^+^ T-cells (154). Therefore, identifying the specific ligand-PAR receptor interactions that mediate beneficial or pathologic effects in different tumor types can aid the development of targeted therapies that avoid unintended pro-tumorigenic effects.

### 5.2 Platelets

Platelets play a significant role in promoting immunosuppressive T-cell phenotypes and impairing the cytotoxic function of NK cells (155). Platelets also promote the development of tumors such as colitis-associated cancer (CAC) by inducing polarization of myeloid cells to MDSCs and reducing the accumulation of CD8^+^ T-cells in the colonic mucosa (156). Inhibiting platelet activation by clopidogrel decreases MDSCs and increases CD8^+^ T-cell infiltration, thereby delaying CAC development (156).

In the circulation, platelets can surround tumor cells, forming tumor microthrombi that are protected from immune surveillance and anoikis (i.e., detachment-triggered apoptosis) (157–160). In the TME, thrombin cleaves glycoprotein A repetitions predominant (GARP) on the platelet surface, liberating surface-bound TGF-ß (161), which activates CAFs to lay down ECM that restricts CD8^+^ T-cells to the periphery of the tumor (162). Platelet-derived TGF-ß also converts effector T-cells to T_regs_ (163, 164). Preventing GARP cleavage, either by inhibiting thrombin or genetically deleting the GARP cleavage site through CRISPR/Cas9 technology, enhances CD8^+^ T cell activation and survival (161).

Tumor-associated platelets also upregulate the surface molecule TLT-1, which promotes CD8^+^ T-cell exhaustion by upregulating checkpoints PD-1 and TIM-3 (165). Furthermore, tumor cells can directly contact platelets in the TME and transfer PD-L1 to them (166, 167). Conversely, platelets can upregulate PD-L1 on ovarian cancer cells through physical contact and indirectly through TGF-β (168). High platelet PD-L1 expression is associated with an immunosuppressive TME by depleting effector T-cells, which lower OS and progression-free survival (PFS) in non-small cell lung cancer patients (NSCLC) (166). Serum levels of platelet PD-L1 might serve as a more accurate indicator of tumor PD-L1 burden and the likelihood of response to ICI therapy compared to the standard immunohistochemical-based quantification of PD-L1 on biopsy specimens (166). Interestingly, PD-L1-expressing platelets may partially explain the efficacy of ICIs even in PD-L1-negative tumors (169). Additionally, PD-L1 was found to activate platelets and amplify thrombosis (170).

### 5.3 Neutrophil extracellular *traps*

Studies have shown that a higher burden of TANs and NETs correlates with reduced T-cell infiltration in the TME, indicating their immunosuppressive effects (171–176). NETs induce exhausted states in CD8^+^ T-cells in murine models (177), and PD-1 within NETs leads to the loss of T-cells by apoptosis (177, 178). Furthermore, NETs have been shown to promote the differentiation of helper T-cells into T_regs_ in murine models of non-alcoholic steatohepatitis (NASH), facilitating the development of NASH-associated HCC (179). Reducing NETs by genetic PAD4-*KO* or Dnase-1 treatment in HCC mice reduces the activity of T_regs_ and enhances anti-tumor NK cell responses (179).

Aside from their direct immunosuppressive effects, NETs also constitute physical barriers that restrict the access of CD8^+^ T-cells and NK cell to tumors (139). In murine models of PDAC, neutrophil recruitment and NETosis induced by IL-17 protect tumor cells against cytotoxic CD8^+^ T-cells, leading to reduced efficacy of immunotherapy (180). Moreover, in radiotherapy treatment for treating bladder cancer, the release of DAMPs such as HMGB1 within dead cell debris triggers NET production through binding TLR4 on the surface of TANs (181). These NETs then create a physical barrier between the tumor and infiltrating CD8^+^ T-cells, reducing anti-tumor immunity (181).

Interestingly, NETs induced by intravesical BCG therapy contribute to anti-tumor immunity by recruiting anti-tumor T-cells and TAMs and enhance therapeutic efficacy of this treatment (182, 183). Tillack et al. showed that NETs primed T-cells by reducing their activation threshold, increasing T-cell responses against specific antigens or even suboptimal stimuli (184). These findings highlight the dual roles of NETs, which can either hinder or augment effective T-cell responses, which may depend on different NET-inducing stimuli, different NET compositions, or perhaps the influence of other, yet undetermined factors in the TME that drive NETs to protective or pathologic functions depending on tumor stage and extent. Hence, exploring the mechanistic aspects of NETs beyond their mere production and physical presence in tumors is crucial. The extrinsic/environmental cues that regulate pro-tumorigenic and anti-tumor phenotypes of NETs remain unknown. Uncovering the specific pathological components of NETs will inform targeted therapeutic strategies mitigating their pathological functions while preserving their beneficial aspects.

## 6 Reprogramming the tumor microenvironment to enhance immunotherapy

### 6.1 Targeting angiogenesis to enhance immunotherapy

Anti-angiogenic therapies were originally developed to inhibit vascular formation, but they inadvertently caused excessive vessel pruning, tumor hypoxia, and decreased anti-tumor immune responses (185, 186). To overcome these limitations, Rakesh Jain proposed the concept of vascular normalization, which involves administering low-dose anti-angiogenic therapy to equilibrate angiogenic signaling within the TME (187). This approach would aim to achieve a state of vascular normalization, characterized by improved tumor blood flow and decreased hypoxia. Numerous studies since have reported findings consistent with the theory of vascular normalization, encapsulated within the idea of a ‘normalization window’ (188). The normalization window represents a brief period of time after administering anti-angiogenic therapy where the tumor vasculature is structurally normalized, during which administered therapeutics can achieve good infiltration into tumor sites (189).

The effectiveness of immunotherapies relies on the infiltration of T-cells into tumor sites in sufficient numbers (190). Tumors that are inflamed and have good immune cell infiltration, referred to as “hot” tumors, exhibit favorable responses to immunotherapies compared to “immune-desert” or “cold” tumors that lack inflammation (190, 191). A normal tumor vasculature, which is not leaky and exhibits a proper pattern of blood flow, is a prerequisite for effective T-cell trafficking into tumor sites.

Thus, vascular normalization with anti-angiogenic drugs can be an effective strategy to enhance therapy response by increasing T-cell infiltration into tumors. Additionally, anti-VEGF medications can counteract the direct immunosuppressive effects of VEGF on various cell types, as discussed earlier (29). Notably, ICIs and anti-angiogenic drugs can synergize in normalizing the tumor vasculature (192), as ICIs can independently improve vessel perfusion, evidenced by improved vessel morphology, increased pericyte coverage, and elevated vessel normalization markers (α-SMA and NG-2) in the TME (192, 193). Therefore, the possibility of combining immunotherapies and anti-angiogenic drugs has garnered significant research interest in recent years, aiming to enhance treatment responses and survival outcomes for cancer patients.

Preclinical studies have demonstrated the potential benefits of combining anti-angiogenic therapies with ICIs, showing increased T-cell infiltration, enhanced local anti-tumor immunity, and improved survival in murine models of different cancer types (194–197). Clinical trials evaluating the efficacy of ICI + anti-angiogenic regimens have shown superior outcomes in various cancers, based on which the FDA has approved the use of these combinations for the treatment of renal cell carcinoma, HCC, NSCLC, and endometrial carcinoma (198–201). We limited our discussion here because other extensive reviews have already been published on this topic (29, 196, 202).

However, combining ICIs and anti-angiogenic therapy has not been effective in highly desmoplastic tumors, such as cholangiocarcinoma, glioblastoma multiforme, and pancreatic adenocarcinoma (203). In these tumors, the dense stroma compresses tumor blood vessels, impeding perfusion and reducing the local delivery of these medications (204). The approach of “stromal normalization” aims to overcome this resistance by reducing stromal density. Angiotensin-converting enzyme (ACE) inhibitors (ACEi) and angiotensin-receptor blockers (ARBs) can inhibit CAFs and reduce ECM production, thus decreasing stromal density and contributing to stromal normalization (204, 205). Clinical studies have shown improved outcomes with anti-VEGF and adjuvant renin-angiotensin system (RAS) inhibitors across tumor types, including glioblastoma, renal cell carcinoma, hepatocellular carcinoma, and metastatic colorectal carcinoma (206–210). However, pancreatic ductal adenocarcinoma remains highly resistant to stromal normalization approaches (211, 212). Recently, a phase-II clinical trial found that adding losartan to chemoradiation therapy for locally advanced unresectable pancreatic cancer resulted in downstaging the tumor and a complete resection rate of 61% (213). This was recently shown to be due to losartan enhancing CD8^+^ T-cell infiltration and decreasing T_regs_ in the TME and reducing immunosuppressive FoxP3^+^ cancer cells, thus enhancing anti-tumor immunity and tumor cell killing (214).

### 6.2 Targeting CAT to enhance immunotherapy

Another potential synergistic approach to reprogram the TME is combining ICIs with anticoagulants. Inhibiting TF has been shown to reduce tumor survival across many *in vitro* studies (215–217). Dabigatran, a thrombin inhibitor, can restrict tumor growth and modify the TME in favor of anti-tumor immunity (218). Rivaroxaban and low-molecular-weight heparin can limit tumor metastasis in mice fibrosarcoma models (145), with rivaroxaban additionally amplifying cytotoxic T-cell responses and stimulating antigen-presenting cells by modulating the FXa-PAR2 axis (145).

The higher risk of thrombotic events associated with ICIs is another compelling reason for combining anticoagulants with ICIs, albeit the mechanisms behind this association remain unclear A retrospective study of 2854 patients receiving ICI found a four-fold increase in the risk of VTE after starting ICI therapy (219). Similarly, a large pharmacovigilance study identified a strong association between the use of ICI and thrombotic complications (220). Combining anticoagulants with immunotherapies could potentially mitigate this risk.

However, clinical evidence for the synergistic effects of ICIs and anticoagulants is conflicting. For instance, Nichetti et al. investigated the impact of the synergistic combination of anti-PD-L1 therapy and anti-platelet agents amongst NSCLC patients, concluding that this synergetic combination did not significantly improve PFS or OS in the multivariate analysis (221). A large study of 728 patients with advanced malignancies found no difference in OS and disease-free survival (DFS) when synergistically combining ICIs and various anticoagulants (including apixaban/rivaroxaban, dabigatran, heparin, and warfarin) (222). However, a recent retrospective study on 280 patients with advanced melanoma demonstrated that treatment with fXa inhibitors enhances the effects of ICIs and confers statistically significant superior PFS and OS (223).

In summary, while preclinical evidence suggests a crucial role for coagulation in fostering an immunosuppressive TME, clinical studies investigating the efficacy of combining anticoagulants and ICIs have yielded varying results. It is essential to recognize that the coagulome of malignant tumors differs significantly across tumor types (224). Interestingly, tumors with highly pro-coagulant properties, such as glioblastoma multiforme and pancreatic adenocarcinomas, are often resistant to ICIs (225). The complex interplay between the TME and coagulome needs further research to better appreciate the impact of targeting the coagulome on TME normalization. Studies into the therapeutically relevant variations in the coagulome among different tumor types are needed. Rigorous clinical trials encompassing different tumor subtypes are required to evaluate the impact of combining anticoagulants and ICIs on tumor progression and patient survival in order to substantiate the encouraging preclinical data.

## 7 Concluding remarks

Several factors need further exploration to improve the effectiveness of ICI + anti-angiogenic regimens, including the optimal dosing and duration of treatment for anti-angiogenic therapy across different tumor types, the underlying mechanisms driving therapeutic responses and resistance, the identification of predictive biomarkers enabling appropriate patient selection and effective therapy response monitoring, and optimizing drug delivery systems. An excellent recent review by Cao et al. covered in detail the applications of anti-angiogenic drugs in cancer and associated challenges (10). Despite the remarkable evolution of these drugs from bench to bedside, survival benefit compared to conventional therapies remains incremental, hence the need for combinatorial approaches (10).

Despite tremendous basic science progress, clinical data on the efficacy of combinatorial approaches, particularly regarding anticoagulation and ICI therapy, are conflicting. The heterogeneity of the coagulome across various tumor types needs to be considered if anticoagulants and ICI combinations are to be furthered. Different components of the coagulome are related to different aspects of the tumor. For instance, levels of TF are closely related to the tumor type, whereas fibrinolysis is highly dependent on TME components (224). The spatial and temporal heterogeneity of the TME is also poorly understood, thereby contributing to discrepant findings attributing both pro-tumor and anti-tumor functions to various components in the TME. Such uncertainties confound translational efforts aimed at targeting these mediators, as there is a risk of inadvertently augmenting pro-tumorigenic processes. We find it likely that, given profound TME heterogeneity across tumor types, future studies will pave the way for a more personalized assessment of patient coagulation status, in line with the major trend of precision medicine in oncology practice.

## Author contributions

AS conceptualized the manuscript. AS, MO, EA, AM, and EI prepared the initial draft and designed the figures. AS, KA, and AY reviewed the manuscript and prepared the final version. All authors contributed to the article and approved the submitted version.

## References

[1] Hanahan D Folkman J. Patterns and emerging mechanisms of the angiogenic switch during tumorigenesis. Cell. (1996) 86(3):353–64. doi: 10.1016/S0092-8674(00)80108-7 8756718

[2] DeBerardinis RJ Chandel NS. We need to talk about the warburg effect. Nat Metab (2020) 2(2):127–9. doi: 10.1038/s42255-020-0172-2 32694689

[3] Mohlin S Wigerup C Jögi A Påhlman S. Hypoxia, pseudohypoxia and cellular differentiation. Exp Cell Res (2017) 356(2):192–6. doi: 10.1016/j.yexcr.2017.03.007 28284840

[4] Schito L Semenza GL. Hypoxia-inducible factors: master regulators of cancer progression. Trends Cancer. (2016) 2(12):758–70. doi: 10.1016/j.trecan.2016.10.016 28741521

[5] Longo DL Bartoli A Consolino L Bardini P Arena F Schwaiger M. *In vivo* imaging of tumor metabolism and acidosis by combining PET and MRI-CEST pH imaging. Cancer Res (2016) 76(22):6463–70. doi: 10.1158/0008-5472.CAN-16-0825 27651313

[6] Helmlinger G Yuan F Dellian M Jain RK. Interstitial pH and pO2 gradients in solid tumors *in vivo*: high-resolution measurements reveal a lack of correlation. Nat Med (1997) 3(2):177–82. doi: 10.1038/nm0297-177 9018236

[7] Chiche J Ilc K Laferrière J Trottier E Dayan F Mazure NM. Hypoxia-inducible carbonic anhydrase IX and XII promote tumor cell growth by counteracting acidosis through the regulation of the intracellular pH. Cancer Res (2009) 69(1):358–68. doi: 10.1158/0008-5472.CAN-08-2470 19118021

[8] Lee P Chandel NS Simon MC. Cellular adaptation to hypoxia through hypoxia inducible factors and beyond. Nat Rev Mol Cell Biol (2020) 21(5):268–83. doi: 10.1038/s41580-020-0227-y PMC7222024 32144406

[9] Liao D Johnson RS. Hypoxia: a key regulator of angiogenesis in cancer. Cancer Metastasis Rev (2007) 26(2):281–90. doi: 10.1007/s10555-007-9066-y 17603752

[10] Cao Y Langer R Ferrara N. Targeting angiogenesis in oncology, ophthalmology and beyond. Nat Rev Drug Discovery. (2023) 22(6):476–95. doi: 10.1038/s41573-023-00671-z 37041221

[11] Ferrara N. Vascular endothelial growth factor: basic science and clinical progress. Endocr Rev (2004) 25(4):581–611. doi: 10.1210/er.2003-0027 15294883

[12] Carmeliet P. VEGF as a key mediator of angiogenesis in cancer. Oncology. (2005) 69 Suppl 3:4–10. doi: 10.1159/000088478 16301830

[13] McDonald DM Baluk P. Imaging of angiogenesis in inflamed airways and tumors: newly formed blood vessels are not alike and may be wildly abnormal: Parker b. Francis lecture. Chest. (2005) 128(6 Suppl):602s–8s. doi: 10.1378/chest.128.6_suppl.602S-a 16373858

[14] Hida K Hida Y Shindoh M. Understanding tumor endothelial cell abnormalities to develop ideal anti-angiogenic therapies. Cancer Sci (2008) 99(3):459–66. doi: 10.1111/j.1349-7006.2007.00704.x PMC11159852 18167133

[15] Hashizume H Baluk P Morikawa S McLean JW Thurston G Roberge S. Openings between defective endothelial cells explain tumor vessel leakiness. Am J Pathol (2000) 156(4):1363–80. doi: 10.1016/S0002-9440(10)65006-7 PMC1876882 10751361

[16] Esser S Wolburg K Wolburg H Breier G Kurzchalia T Risau W. Vascular endothelial growth factor induces endothelial fenestrations *in vitro* . J Cell Biol (1998) 140(4):947–59. doi: 10.1083/jcb.140.4.947 PMC2141756 9472045

[17] Bergers G Song S. The role of pericytes in blood-vessel formation and maintenance. Neuro Oncol (2005) 7(4):452–64. doi: 10.1215/S1152851705000232 PMC1871727 16212810

[18] Franco M Roswall P Cortez E Hanahan D Pietras K. Pericytes promote endothelial cell survival through induction of autocrine VEGF-a signaling and bcl-w expression. Blood. (2011) 118(10):2906–17. doi: 10.1182/blood-2011-01-331694 PMC3172806 21778339

[19] Morikawa S Baluk P Kaidoh T Haskell A Jain RK McDonald DM. Abnormalities in pericytes on blood vessels and endothelial sprouts in tumors. Am J Pathol (2002) 160(3):985–1000. doi: 10.1016/S0002-9440(10)64920-6 11891196 PMC1867175

[20] Chang J Chaudhuri O. Beyond proteases: basement membrane mechanics and cancer invasion. J Cell Biol (2019) 218(8):2456–69. doi: 10.1083/jcb.201903066 PMC6683740 31315943

[21] Piali L Fichtel A Terpe HJ Imhof BA Gisler RH. Endothelial vascular cell adhesion molecule 1 expression is suppressed by melanoma and carcinoma. J Exp Med (1995) 181(2):811–6. doi: 10.1084/jem.181.2.811 PMC2191895 7530765

[22] Tabruyn SP Sabatel C Nguyen NQ Verhaeghe C Castermans K Malvaux L. The angiostatic 16K human prolactin overcomes endothelial cell anergy and promotes leukocyte infiltration *via* nuclear factor-kappaB activation. Mol Endocrinol (2007) 21(6):1422–9. doi: 10.1210/me.2007-0021 17405903

[23] Huang H Langenkamp E Georganaki M Loskog A Fuchs PF Dieterich LC. VEGF suppresses T-lymphocyte infiltration in the tumor microenvironment through inhibition of NF-κB-induced endothelial activation. FASEB J (2015) 29(1):227–38. doi: 10.1096/fj.14-250985 25361735

[24] Dirkx AE oude Egbrink MG Castermans K van der Schaft DW Thijssen VL Dings RP. Anti-angiogenesis therapy can overcome endothelial cell anergy and promote leukocyte-endothelium interactions and infiltration in tumors. FASEB J (2006) 20(6):621–30. doi: 10.1096/fj.05-4493com 16581970

[25] Ma W Wang Y Zhang R Yang F Zhang D Huang M. Targeting PAK4 to reprogram the vascular microenvironment and improve CAR-T immunotherapy for glioblastoma. Nat Cancer. (2021) 2(1):83–97. doi: 10.1038/s43018-020-00147-8 35121889 PMC10097424

[26] Motz GT Santoro SP Wang LP Garrabrant T Lastra RR Hagemann IS. Tumor endothelium FasL establishes a selective immune barrier promoting tolerance in tumors. Nat Med (2014) 20(6):607–15. doi: 10.1038/nm.3541 PMC4060245 24793239

[27] Liu S Qin T Liu Z Wang J Jia Y Feng Y. Anlotinib alters tumor immune microenvironment by downregulating PD-L1 expression on vascular endothelial cells. Cell Death Disease. (2020) 11(5):309. doi: 10.1038/s41419-020-2511-3 32366856 PMC7198575

[28] Fukumura D Kloepper J Amoozgar Z Duda DG Jain RK. Enhancing cancer immunotherapy using antiangiogenics: opportunities and challenges. Nat Rev Clin Oncol (2018) 15(5):325–40. doi: 10.1038/nrclinonc.2018.29 PMC5921900 29508855

[29] Lee WS Yang H Chon HJ Kim C. Combination of anti-angiogenic therapy and immune checkpoint blockade normalizes vascular-immune crosstalk to potentiate cancer immunity. Exp Mol Med (2020) 52(9):1475–85. doi: 10.1038/s12276-020-00500-y PMC8080646 32913278

[30] Voron T Colussi O Marcheteau E Pernot S Nizard M Pointet AL. VEGF-a modulates expression of inhibitory checkpoints on CD8+ T cells in tumors. J Exp Med (2015) 212(2):139–48. doi: 10.1084/jem.20140559 PMC4322048 25601652

[31] Suthen S Lim CJ Nguyen PHD Dutertre CA Lai HLH Wasser M. Hypoxia-driven immunosuppression by treg and type-2 conventional dendritic cells in HCC. Hepatology. (2022) 76(5):1329–44. doi: 10.1002/hep.32419 35184329

[32] Salman S Meyers DJ Wicks EE Lee SN Datan E Thomas AM. HIF inhibitor 32-134D eradicates murine hepatocellular carcinoma in combination with anti-PD1 therapy. J Clin Invest. (2022) 132(9):e156774. doi: 10.1172/JCI156774 35499076 PMC9057582

[33] Bailey CM Liu Y Liu M Du X Devenport M Zheng P. Targeting HIF-1α abrogates PD-L1–mediated immune evasion in tumor microenvironment but promotes tolerance in normal tissues. J Clin Invest (2022) 132(9):e150846. doi: 10.1172/JCI150846 35239514 PMC9057613

[34] Cowman SJ Fuja DG Liu X-D Tidwell RSS Kandula N Sirohi D. Macrophage HIF-1α is an independent prognostic indicator in kidney cancer. Clin Cancer Res (2020) 26(18):4970–82. doi: 10.1158/1078-0432.CCR-19-3890 PMC7968518 32586940

[35] Chen F Chen J Yang L Liu J Zhang X Zhang Y. Extracellular vesicle-packaged HIF-1α-stabilizing lncRNA from tumour-associated macrophages regulates aerobic glycolysis of breast cancer cells. Nat Cell Biol (2019) 21(4):498–510. doi: 10.1038/s41556-019-0299-0 30936474

[36] Chen B Li L Li M Wang X. HIF1A expression correlates with increased tumor immune and stromal signatures and aggressive phenotypes in human cancers. Cell Oncol (Dordr). (2020) 43(5):877–88. doi: 10.1007/s13402-020-00534-4 PMC12990741 32488852

[37] Wu Q Zhou W Yin S Zhou Y Chen T Qian J. Blocking triggering receptor expressed on myeloid cells-1-Positive tumor-associated macrophages induced by hypoxia reverses immunosuppression and anti-programmed cell death ligand 1 resistance in liver cancer. Hepatology. (2019) 70(1):198–214. doi: 10.1002/hep.30593 30810243 PMC6618281

[38] Giatromanolaki A Koukourakis MI Sivridis E Turley H Talks K Pezzella F. Relation of hypoxia inducible factor 1 alpha and 2 alpha in operable non-small cell lung cancer to angiogenic/molecular profile of tumours and survival. Br J Cancer. (2001) 85(6):881–90. doi: 10.1054/bjoc.2001.2018 PMC2375073 11556841

[39] Maranchie JK Vasselli JR Riss J Bonifacino JS Linehan WM Klausner RD. The contribution of VHL substrate binding and HIF1-alpha to the phenotype of VHL loss in renal cell carcinoma. Cancer Cell (2002) 1(3):247–55. doi: 10.1016/S1535-6108(02)00044-2 12086861

[40] Imtiyaz HZ Williams EP Hickey MM Patel SA Durham AC Yuan L-J. Hypoxia-inducible factor 2α regulates macrophage function in mouse models of acute and tumor inflammation. J Clin Invest (2010) 120(8):2699–714. doi: 10.1172/JCI39506 PMC2912179 20644254

[41] Steinberger KJ Eubank TD. The Underexplored Landscape of Hypoxia-Inducible Factor 2 Alpha and Potential Roles in Tumor Macrophages: A Review. Oxygen (Basel) (2023) 3(1):45–76. doi: 10.3390/oxygen3010005 37124241 PMC10137047

[42] McFarlane AJ Fercoq F Coffelt SB Carlin LM. Neutrophil dynamics in the tumor microenvironment. J Clin Invest (2021) 131(6):e143759. doi: 10.1172/JCI143759 33720040 PMC7954585

[43] Walmsley SR Print C Farahi N Peyssonnaux C Johnson RS Cramer T. Hypoxia-induced neutrophil survival is mediated by HIF-1alpha-dependent NF-kappaB activity. J Exp Med (2005) 201(1):105–15. doi: 10.1084/jem.20040624 PMC2212759 15630139

[44] Thompson AA Elks PM Marriott HM Eamsamarng S Higgins KR Lewis A. Hypoxia-inducible factor 2α regulates key neutrophil functions in humans, mice, and zebrafish. Blood. (2014) 123(3):366–76. doi: 10.1182/blood-2013-05-500207 PMC3894493 24196071

[45] McGovern NN Cowburn AS Porter L Walmsley SR Summers C Thompson AAR. Hypoxia selectively inhibits respiratory burst activity and killing of staphylococcus aureus in human neutrophils. J Immunol (2011) 186(1):453–63. doi: 10.4049/jimmunol.1002213 PMC4374781 21135168

[46] Corzo CA Condamine T Lu L Cotter MJ Youn JI Cheng P. HIF-1α regulates function and differentiation of myeloid-derived suppressor cells in the tumor microenvironment. J Exp Med (2010) 207(11):2439–53. doi: 10.1084/jem.20100587 PMC2964584 20876310

[47] Lin N Simon MC. Hypoxia-inducible factors: key regulators of myeloid cells during inflammation. J Clin Invest. (2016) 126(10):3661–71. doi: 10.1172/JCI84426 PMC5096831 27599290

[48] Mahiddine K Blaisdell A Ma S Créquer-Grandhomme A Lowell CA Erlebacher A. Relief of tumor hypoxia unleashes the tumoricidal potential of neutrophils. J Clin Invest (2020) 130(1):389–403. doi: 10.1172/JCI130952 31600172 PMC6934192

[49] Long J Hu Z Xue H Wang Y Chen J Tang F. Vascular endothelial growth factor (VEGF) impairs the motility and immune function of human mature dendritic cells through the VEGF receptor 2-RhoA-cofilin1 pathway. Cancer Sci (2019) 110(8):2357–67. doi: 10.1111/cas.14091 PMC6676124 31169331

[50] Gabrilovich DI Chen HL Girgis KR Cunningham HT Meny GM Nadaf S. Production of vascular endothelial growth factor by human tumors inhibits the functional maturation of dendritic cells. Nat Med (1996) 2(10):1096–103. doi: 10.1038/nm1096-1096 8837607

[51] Kortylewski M Kujawski M Wang T Wei S Zhang S Pilon-Thomas S. Inhibiting Stat3 signaling in the hematopoietic system elicits multicomponent antitumor immunity. Nat Med (2005) 11(12):1314–21. doi: 10.1038/nm1325 16288283

[52] Pang L Ng KT-P Liu J Yeung W-HO Zhu J Chiu T-LS. Plasmacytoid dendritic cells recruited by HIF-1α/eADO/ADORA1 signaling induce immunosuppression in hepatocellular carcinoma. Cancer Letters. (2021) 522:80–92. doi: 10.1016/j.canlet.2021.09.022 34536555

[53] Akkaya B Oya Y Akkaya M Al Souz J Holstein AH Kamenyeva O. Regulatory T cells mediate specific suppression by depleting peptide–MHC class II from dendritic cells. Nat Immunol (2019) 20(2):218–31. doi: 10.1038/s41590-018-0280-2 PMC6402611 30643268

[54] Paardekooper LM Vos W van den Bogaart G. Oxygen in the tumor microenvironment: effects on dendritic cell function. Oncotarget. (2019) 10(8):883–96. doi: 10.18632/oncotarget.26608 PMC6368231 30783517

[55] Iwanowycz S Ngoi S Li Y Hill M Koivisto C Parrish M. Type 2 dendritic cells mediate control of cytotoxic T cell resistant tumors. JCI Insight (2021) 6(17):e145885. doi: 10.1172/jci.insight.145885 34283809 PMC8492342

[56] Maier B Leader AM Chen ST Tung N Chang C LeBerichel J. A conserved dendritic-cell regulatory program limits antitumour immunity. Nature. (2020) 580(7802):257–62. doi: 10.1038/s41586-020-2134-y PMC7787191 32269339

[57] Clambey ET McNamee EN Westrich JA Glover LE Campbell EL Jedlicka P. Hypoxia-inducible factor-1 alpha-dependent induction of FoxP3 drives regulatory T-cell abundance and function during inflammatory hypoxia of the mucosa. Proc Natl Acad Sci U S A. (2012) 109(41):E2784–93. doi: 10.1073/pnas.1202366109 PMC3478644 22988108

[58] Hasmim M Noman MZ Messai Y Bordereaux D Gros G Baud V. Cutting edge: hypoxia-induced nanog favors the intratumoral infiltration of regulatory T cells and macrophages *via* direct regulation of TGF-β1. J Immunol (2013) 191(12):5802–6. doi: 10.4049/jimmunol.1302140 24227785

[59] Facciabene A Peng X Hagemann IS Balint K Barchetti A Wang LP. Tumour hypoxia promotes tolerance and angiogenesis *via* CCL28 and t(reg) cells. Nature. (2011) 475(7355):226–30. doi: 10.1038/nature10169 21753853

[60] Scharping NE Rivadeneira DB Menk AV Vignali PDA Ford BR Rittenhouse NL. Mitochondrial stress induced by continuous stimulation under hypoxia rapidly drives T cell exhaustion. Nat Immunol (2021) 22(2):205–15. doi: 10.1038/s41590-020-00834-9 PMC7971090 33398183

[61] Sitkovsky MV Kjaergaard J Lukashev D Ohta A. Hypoxia-adenosinergic immunosuppression: tumor protection by T regulatory cells and cancerous tissue hypoxia. Clin Cancer Res (2008) 14(19):5947–52. doi: 10.1158/1078-0432.CCR-08-0229 18829471

[62] Vignali PDA DePeaux K Watson MJ Ye C Ford BR Lontos K. Hypoxia drives CD39-dependent suppressor function in exhausted T cells to limit antitumor immunity. Nat Immunol (2023) 24(2):267–79. doi: 10.1038/s41590-022-01379-9 PMC10402660 36543958

[63] Doedens AL Phan AT Stradner MH Fujimoto JK Nguyen JV Yang E. Hypoxia-inducible factors enhance the effector responses of CD8(+) T cells to persistent antigen. Nat Immunol (2013) 14(11):1173–82. doi: 10.1038/ni.2714 PMC3977965 24076634

[64] Tyrakis PA Palazon A Macias D Lee KL Phan AT Veliça P. S-2-hydroxyglutarate regulates CD8(+) T-lymphocyte fate. Nature. (2016) 540(7632):236–41. doi: 10.1038/nature20165 PMC5149074 27798602

[65] Nanduri J Wang N Yuan G Khan SA Souvannakitti D Peng Y-J. Intermittent hypoxia degrades HIF-2α *via* calpains resulting in oxidative stress: implications for recurrent apnea-induced morbidities. Proc Natl Acad Sci (2009) 106(4):1199–204. doi: 10.1073/pnas.0811018106 PMC2626608 19147445

[66] Chen L Gao Y Li Y Wang C Chen D Gao Y. Severe intermittent hypoxia modulates the macrophage phenotype and impairs wound healing through downregulation of HIF-2α. Nat Sci Sleep. (2022) 14:1511–20. doi: 10.2147/NSS.S382275 PMC9441177 36068885

[67] Sahai E Astsaturov I Cukierman E DeNardo DG Egeblad M Evans RM. A framework for advancing our understanding of cancer-associated fibroblasts. Nat Rev Cancer. (2020) 20(3):174–86. doi: 10.1038/s41568-019-0238-1 PMC7046529 31980749

[68] Kato T Noma K Ohara T Kashima H Katsura Y Sato H. Cancer-associated fibroblasts affect intratumoral CD8+ and FoxP3+ T cells *Via* IL6 in the tumor microenvironment. Clin Cancer Res (2018) 24(19):4820–33. doi: 10.1158/1078-0432.CCR-18-0205 29921731

[69] Nishiwaki N Noma K Ohara T Kunitomo T Kawasaki K Akai M. Overcoming cancer-associated fibroblast-induced immunosuppression by anti-interleukin-6 receptor antibody. Cancer Immunology Immunother (2023) 72(7):2029–2044. doi: 10.1007/s00262-023-03378-7 PMC9916502 36764954

[70] Bohn T Rapp S Luther N Klein M Bruehl T-J Kojima N. Tumor immunoevasion *via* acidosis-dependent induction of regulatory tumor-associated macrophages. Nat Immunol (2018) 19(12):1319–29. doi: 10.1038/s41590-018-0226-8 30397348

[71] Ohashi T Aoki M Tomita H Akazawa T Sato K Kuze B. M2-like macrophage polarization in high lactic acid-producing head and neck cancer. Cancer Science. (2017) 108(6):1128–34. doi: 10.1111/cas.13244 PMC5480089 28370718

[72] Gao X Zhou S Qin Z Li D Zhu Y Ma D. Upregulation of HMGB1 in tumor-associated macrophages induced by tumor cell-derived lactate further promotes colorectal cancer progression. J Trans Med (2023) 21(1):53. doi: 10.1186/s12967-023-03918-w PMC9883966 36709284

[73] Colegio OR Chu N-Q Szabo AL Chu T Rhebergen AM Jairam V. Functional polarization of tumour-associated macrophages by tumour-derived lactic acid. Nature. (2014) 513(7519):559–63. doi: 10.1038/nature13490 PMC4301845 25043024

[74] Fischer K Hoffmann P Voelkl S Meidenbauer N Ammer J Edinger M. Inhibitory effect of tumor cell–derived lactic acid on human T cells. Blood. (2007) 109(9):3812–9. doi: 10.1182/blood-2006-07-035972 17255361

[75] Brand A Singer K Koehl Gudrun E Kolitzus M Schoenhammer G Thiel A. LDHA-associated lactic acid production blunts tumor immunosurveillance by T and NK cells. Cell Metab (2016) 24(5):657–71. doi: 10.1016/j.cmet.2016.08.011 27641098

[76] Pilon-Thomas S Kodumudi KN El-Kenawi AE Russell S Weber AM Luddy K. Correction: neutralization of tumor acidity improves antitumor responses to immunotherapy. Cancer Res (2017) 77(9):2552. doi: 10.1158/0008-5472.CAN-15-1743 28461565

[77] Fischbeck AJ Ruehland S Ettinger A Paetzold K Masouris I Noessner E. Tumor lactic acidosis: protecting tumor by inhibiting cytotoxic activity through motility arrest and bioenergetic silencing. Front Oncol (2020) 10. doi: 10.3389/fonc.2020.589434 PMC7753121 33364193

[78] Haas R Smith J Rocher-Ros V Nadkarni S Montero-Melendez T D’Acquisto F. Lactate regulates metabolic and pro-inflammatory circuits in control of T cell migration and effector functions. PloS Biol (2015) 13(7):e1002202. doi: 10.1371/journal.pbio.1002202 26181372 PMC4504715

[79] Bosticardo M Ariotti S Losana G Bernabei P Forni G Novelli F. Biased activation of human T lymphocytes due to low extracellular pH is antagonized by B7/CD28 costimulation. Eur J Immunol (2001) 31(9):2829–38. doi: 10.1002/1521-4141(200109)31:9<2829::AID-IMMU2829>3.0.CO;2-U 11536182

[80] Pilon-Thomas S Kodumudi KN El-Kenawi AE Russell S Weber AM Luddy K. Neutralization of tumor acidity improves antitumor responses to immunotherapy. Cancer Res (2016) 76(6):1381–90. doi: 10.1158/0008-5472.CAN-15-1743 PMC4829106 26719539

[81] Ho P-C Bihuniak Jessica D Macintyre Andrew N Staron M Liu X Amezquita R. Phosphoenolpyruvate is a metabolic checkpoint of anti-tumor T cell responses. Cell. (2015) 162(6):1217–28. doi: 10.1016/j.cell.2015.08.012 PMC4567953 26321681

[82] Scharping NE Menk AV Moreci RS Whetstone RD Dadey RE Watkins SC. The tumor microenvironment represses T cell mitochondrial biogenesis to drive intratumoral T cell metabolic insufficiency and dysfunction. Immunity. (2016) 45(2):374–88. doi: 10.1016/j.immuni.2016.07.009 PMC5207350 27496732

[83] Najjar YG Menk AV Sander C Rao U Karunamurthy A Bhatia R. Tumor cell oxidative metabolism as a barrier to PD-1 blockade immunotherapy in melanoma. JCI Insight (2019) 4(5):e124989. doi: 10.1172/jci.insight.124989 30721155 PMC6483505

[84] Zheng Y Delgoffe GM Meyer CF Chan W Powell JD. Anergic T cells are metabolically Anergic1. J Immunol (2009) 183(10):6095–101. doi: 10.4049/jimmunol.0803510 PMC2884282 19841171

[85] Vander Heiden MG Cantley LC Thompson CB. Understanding the warburg effect: the metabolic requirements of cell proliferation. Science. (2009) 324(5930):1029–33. doi: 10.1126/science.1160809 PMC2849637 19460998

[86] Chang C-H Qiu J O’Sullivan D Buck Michael D Noguchi T Curtis Jonathan D. Metabolic competition in the tumor microenvironment is a driver of cancer progression. Cell. (2015) 162(6):1229–41. doi: 10.1016/j.cell.2015.08.016 PMC4864363 26321679

[87] Pearce Erika L Pearce Edward J. Metabolic pathways in immune cell activation and quiescence. Immunity. (2013) 38(4):633–43. doi: 10.1016/j.immuni.2013.04.005 PMC3654249 23601682

[88] Faubert B Li KY Cai L Hensley CT Kim J Zacharias LG. Lactate metabolism in human lung tumors. Cell. (2017) 171(2):358–71.e9. doi: 10.1016/j.cell.2017.09.019 28985563 PMC5684706

[89] Boedtkjer E Pedersen SF. The acidic tumor microenvironment as a driver of cancer. Annu Rev Physiol (2020) 82(1):103–26. doi: 10.1146/annurev-physiol-021119-034627 31730395

[90] Thommen DS Schumacher TN. T Cell dysfunction in cancer. Cancer Cell (2018) 33(4):547–62. doi: 10.1016/j.ccell.2018.03.012 PMC7116508 29634943

[91] Wang H Franco F Tsui Y-C Xie X Trefny MP Zappasodi R. CD36-mediated metabolic adaptation supports regulatory T cell survival and function in tumors. Nat Immunol (2020) 21(3):298–308. doi: 10.1038/s41590-019-0589-5 32066953 PMC7043937

[92] Michalek RD Gerriets VA Jacobs SR Macintyre AN MacIver NJ Mason EF. Cutting edge: distinct glycolytic and lipid oxidative metabolic programs are essential for effector and regulatory CD4+ T cell subsets. J Immunol (2011) 186(6):3299–303. doi: 10.4049/jimmunol.1003613 PMC3198034 21317389

[93] Watson MJ Vignali PDA Mullett SJ Overacre-Delgoffe AE Peralta RM Grebinoski S. Metabolic support of tumour-infiltrating regulatory T cells by lactic acid. Nature. (2021) 591(7851):645–51. doi: 10.1038/s41586-020-03045-2 PMC7990682 33589820

[94] Zappasodi R Serganova I Cohen IJ Maeda M Shindo M Senbabaoglu Y. CTLA-4 blockade drives loss of treg stability in glycolysis-low tumours. Nature. (2021) 591(7851):652–8. doi: 10.1038/s41586-021-03326-4 PMC8057670 33588426

[95] Kim MJ Kim K Park HJ Kim G-R Hong KH Oh JH. Deletion of PD-1 destabilizes the lineage identity and metabolic fitness of tumor-infiltrating regulatory T cells. Nat Immunol (2023) 24(1):148–61. doi: 10.1038/s41590-022-01373-1 36577929

[96] Feng Q Liu Z Yu X Huang T Chen J Wang J. Lactate increases stemness of CD8 + T cells to augment anti-tumor immunity. Nat Commun (2022) 13(1):4981. doi: 10.1038/s41467-022-32521-8 36068198 PMC9448806

[97] Cheng H Qiu Y Xu Y Chen L Ma K Tao M. Extracellular acidosis restricts one-carbon metabolism and preserves T cell stemness. Nat Metab (2023) 5(2):314–30. doi: 10.1038/s42255-022-00730-6 PMC9970874 36717749

[98] JW BLOM Vanderschoot J Oostindier M OSANTO S van der Meer F Rosendaal F. Incidence of venous thrombosis in a large cohort of 66 329 cancer patients: results of a record linkage study. J Thromb Haemostasis. (2006) 4(3):529–35. doi: 10.1111/j.1538-7836.2006.01804.x 16460435

[99] Sørensen HT Mellemkjær L Olsen JH Baron JA. Prognosis of cancers associated with venous thromboembolism. New Engl J Med (2000) 343(25):1846–50. doi: 10.1056/NEJM200012213432504 11117976

[100] Khorana AA Francis CW Culakova E Kuderer NM Lyman GH. Thromboembolism is a leading cause of death in cancer patients receiving outpatient chemotherapy. J Thromb Haemost. (2007) 5(3):632–4. doi: 10.1111/j.1538-7836.2007.02374.x 17319909

[101] Abu Saadeh F Norris L O’Toole S Mohamed BM Langhe R O’Leary J. Tumour expresion of tissue factor and tissue factor pathway inhibitor in ovarian cancer- relationship with venous thrombosis risk. Thromb Res (2013) 132(5):627–34. doi: 10.1016/j.thromres.2013.09.016 24094893

[102] Uno K Homma S Satoh T Nakanishi K Abe D Matsumoto K. Tissue factor expression as a possible determinant of thromboembolism in ovarian cancer. Br J Cancer. (2007) 96(2):290–5. doi: 10.1038/sj.bjc.6603552 PMC2359996 17211468

[103] Khorana AA Kamphuisen PW Meyer G Bauersachs R Janas MS Jarner MF. Tissue factor as a predictor of recurrent venous thromboembolism in malignancy: biomarker analyses of the CATCH trial. J Clin Oncol (2016) 35(10):1078–85. doi: 10.1200/JCO.2016.67.4564 28029329

[104] Campello E Ilich A Simioni P Key NS. The relationship between pancreatic cancer and hypercoagulability: a comprehensive review on epidemiological and biological issues. Br J Cancer. (2019) 121(5):359–71. doi: 10.1038/s41416-019-0510-x PMC6738049 31327867

[105] van Es N Hisada Y Di Nisio M Cesarman G Kleinjan A Mahé I. Extracellular vesicles exposing tissue factor for the prediction of venous thromboembolism in patients with cancer: a prospective cohort study. Thromb Res (2018) 166:54–9. doi: 10.1016/j.thromres.2018.04.009 29656167

[106] Mackman N Sachetto ATA Hisada Y. Measurement of tissue factor-positive extracellular vesicles in plasma: strengths and weaknesses of current methods. Curr Opin Hematol (2022) 29(5):266–74. doi: 10.1097/MOH.0000000000000730 35852819

[107] Mitrugno A Tormoen GW Kuhn P McCarty OJT. The prothrombotic activity of cancer cells in the circulation. Blood Rev (2016) 30(1):11–9. doi: 10.1016/j.blre.2015.07.001 PMC4942124 26219246

[108] Unruh D Horbinski C. Beyond thrombosis: the impact of tissue factor signaling in cancer. J Hematol Oncol (2020) 13(1):93. doi: 10.1186/s13045-020-00932-z 32665005 PMC7362520

[109] Hu Z. Tissue factor as a new target for CAR-NK cell immunotherapy of triple-negative breast cancer. Sci Rep (2020) 10(1):2815. doi: 10.1038/s41598-020-59736-3 32071339 PMC7028910

[110] Cantrell R Palumbo JS. Hemostasis and tumor immunity. Res Pract Thromb Haemost. (2022) 6(4):e12728. doi: 10.1002/rth2.12728 35647476 PMC9130907

[111] Wang X Zhao S Wang Z Gao T. Platelets involved tumor cell EMT during circulation: communications and interventions. Cell Communication Signaling (2022) 20(1):82. doi: 10.1186/s12964-022-00887-3 35659308 PMC9166407

[112] Wang L Wang X Guo E Mao X Miao S. Emerging roles of platelets in cancer biology and their potential as therapeutic targets. Front Oncol (2022) 12:939089. doi: 10.3389/fonc.2022.939089 35936717 PMC9355257

[113] Suzuki-Inoue K. Platelets and cancer-associated thrombosis: focusing on the platelet activation receptor CLEC-2 and podoplanin. Hematology. (2019) 2019(1):175–81. doi: 10.1182/hematology.2019001388 PMC6913448 31808911

[114] Suzuki-Inoue K Kato Y Inoue O Kaneko MK Mishima K Yatomi Y. Involvement of the snake toxin receptor CLEC-2, in podoplanin-mediated platelet activation, by cancer cells. J Biol Chem (2007) 282(36):25993–6001. doi: 10.1074/jbc.M702327200 17616532

[115] Takagi S Sato S Oh-hara T Takami M Koike S Mishima Y. Platelets promote tumor growth and metastasis *via* direct interaction between Aggrus/Podoplanin and CLEC-2. PloS One (2013) 8(8):e73609. doi: 10.1371/journal.pone.0073609 23991201 PMC3749157

[116] Geddings JE Hisada Y Boulaftali Y Getz TM Whelihan M Fuentes R. Tissue factor-positive tumor microvesicles activate platelets and enhance thrombosis in mice. J Thromb Haemost. (2016) 14(1):153–66. doi: 10.1111/jth.13181 PMC4715578 26516108

[117] Zarà M Canobbio I Visconte C Canino J Torti M Guidetti GF. Molecular mechanisms of platelet activation and aggregation induced by breast cancer cells. Cell Signal (2018) 48:45–53. doi: 10.1016/j.cellsig.2018.04.008 29705335

[118] McNamee N de la Fuente LR Santos-Martinez MJ O’Driscoll L. Proteomics profiling identifies extracellular vesicles’ cargo associated with tumour cell induced platelet aggregation. BMC Cancer. (2022) 22(1):1023. doi: 10.1186/s12885-022-10068-7 36171564 PMC9520807

[119] Tesfamariam B. Involvement of platelets in tumor cell metastasis. Pharmacol Ther (2016) 157:112–9. doi: 10.1016/j.pharmthera.2015.11.005 26615781

[120] Gay LJ Felding-Habermann B. Contribution of platelets to tumour metastasis. Nat Rev Cancer. (2011) 11(2):123–34. doi: 10.1038/nrc3004 PMC6894505 21258396

[121] Franco AT Corken A Ware J. Platelets at the interface of thrombosis, inflammation, and cancer. Blood. (2015) 126(5):582–8. doi: 10.1182/blood-2014-08-531582 PMC4520875 26109205

[122] Papayannopoulos V. Neutrophil extracellular traps in immunity and disease. Nat Rev Immunol (2018) 18(2):134–47. doi: 10.1038/nri.2017.105 28990587

[123] Yang C Sun W Cui W Li X Yao J Jia X. Procoagulant role of neutrophil extracellular traps in patients with gastric cancer. Int J Clin Exp Pathol (2015) 8(11):14075–86. PMC4713507 26823721

[124] Thålin C Demers M Blomgren B Wong SL von Arbin M von Heijne A. NETosis promotes cancer-associated arterial microthrombosis presenting as ischemic stroke with troponin elevation. Thromb Res (2016) 139:56–64. doi: 10.1016/j.thromres.2016.01.009 26916297 PMC4769435

[125] Oklu R Sheth RA Wong KHK Jahromi AH Albadawi H. Neutrophil extracellular traps are increased in cancer patients but does not associate with venous thrombosis. Cardiovasc Diagn Ther (2017) 7(Suppl 3):S140–s9. doi: 10.21037/cdt.2017.08.01 PMC5778521 29399517

[126] Mauracher LM Posch F Martinod K Grilz E Däullary T Hell L. Citrullinated histone H3, a biomarker of neutrophil extracellular trap formation, predicts the risk of venous thromboembolism in cancer patients. J Thromb Haemost. (2018) 16(3):508–18. doi: 10.1111/jth.13951 PMC6294121 29325226

[127] Cedervall J Dragomir A Saupe F Zhang Y Ärnlöv J Larsson E. Pharmacological targeting of peptidylarginine deiminase 4 prevents cancer-associated kidney injury in mice. OncoImmunology. (2017) 6(8):e1320009. doi: 10.1080/2162402X.2017.1320009 28919990 PMC5593702

[128] Cedervall J Zhang Y Huang H Zhang L Femel J Dimberg A. Neutrophil extracellular traps accumulate in peripheral blood vessels and compromise organ function in tumor-bearing animals. Cancer Res (2015) 75(13):2653–62. doi: 10.1158/0008-5472.CAN-14-3299 26071254

[129] Hisada Y Grover SP Maqsood A Houston R Ay C Noubouossie DF. Neutrophils and neutrophil extracellular traps enhance venous thrombosis in mice bearing human pancreatic tumors. Haematologica. (2020) 105(1):218–25. doi: 10.3324/haematol.2019.217083 PMC6939515 31048354

[130] Leal AC Mizurini DM Gomes T Rochael NC Saraiva EM Dias MS. Tumor-derived exosomes induce the formation of neutrophil extracellular traps: implications for the establishment of cancer-associated thrombosis. Sci Rep (2017) 7(1):6438. doi: 10.1038/s41598-017-06893-7 28743887 PMC5526939

[131] Rosell A Martinod K Mackman N Thålin C. Neutrophil extracellular traps and cancer-associated thrombosis. Thromb Res (2022) 213:S35–41. doi: 10.1016/j.thromres.2021.12.018 36210559

[132] Demers M Krause DS Schatzberg D Martinod K Voorhees JR Fuchs TA. Cancers predispose neutrophils to release extracellular DNA traps that contribute to cancer-associated thrombosis. Proc Natl Acad Sci (2012) 109(32):13076–81. doi: 10.1073/pnas.1200419109 PMC3420209 22826226

[133] Xiao Y Cong M Li J He D Wu Q Tian P. Cathepsin c promotes breast cancer lung metastasis by modulating neutrophil infiltration and neutrophil extracellular trap formation. Cancer Cell (2021) 39(3):423–37.e7. doi: 10.1016/j.ccell.2020.12.012 33450198

[134] Yang L Liu L Zhang R Hong J Wang Y Wang J. IL-8 mediates a positive loop connecting increased neutrophil extracellular traps (NETs) and colorectal cancer liver metastasis. J Cancer. (2020) 11(15):4384–96. doi: 10.7150/jca.44215 PMC7255375 32489457

[135] Ortiz-Espinosa S Morales X Senent Y Alignani D Tavira B Macaya I. Complement C5a induces the formation of neutrophil extracellular traps by myeloid-derived suppressor cells to promote metastasis. Cancer Letters. (2022) 529:70–84. doi: 10.1016/j.canlet.2021.12.027 34971753

[136] Yang J Jin L Kim HS Tian F Yi Z Bedi K. KDM6A loss recruits tumor-associated neutrophils and promotes neutrophil extracellular trap formation in pancreatic cancer. Cancer Res (2022) 82(22):4247–60. doi: 10.1158/0008-5472.CAN-22-0968 PMC9669233 36306422

[137] Kowanetz M Wu X Lee J Tan M Hagenbeek T Qu X. Granulocyte-colony stimulating factor promotes lung metastasis through mobilization of Ly6G+Ly6C+ granulocytes. Proc Natl Acad Sci U S A. (2010) 107(50):21248–55. doi: 10.1073/pnas.1015855107 PMC3003076 21081700

[138] Teijeira A Garasa S Ochoa MDC Cirella A Olivera I Glez-Vaz J. Differential interleukin-8 thresholds for chemotaxis and netosis in human neutrophils. Eur J Immunol (2021) 51(9):2274–80. doi: 10.1002/eji.202049029 33963542

[139] Teijeira Á Garasa S Gato M Alfaro C Migueliz I Cirella A. CXCR1 and CXCR2 chemokine receptor agonists produced by tumors induce neutrophil extracellular traps that interfere with immune cytotoxicity. Immunity. (2020) 52(5):856–71.e8. doi: 10.1016/j.immuni.2020.03.001 32289253

[140] Etulain J Martinod K Wong SL Cifuni SM Schattner M Wagner DD. P-selectin promotes neutrophil extracellular trap formation in mice. Blood (2015) 126(2):242–6. doi: 10.1182/blood-2015-01-624023 PMC4497964 25979951

[141] Tadie J-M Bae H-B Jiang S Park DW Bell CP Yang H. HMGB1 promotes neutrophil extracellular trap formation through interactions with toll-like receptor 4. Am J Physiology-Lung Cell Mol Physiol (2013) 304(5):L342–L9. doi: 10.1152/ajplung.00151.2012 PMC3602738 23316068

[142] Dyer MR Chen Q Haldeman S Yazdani H Hoffman R Loughran P. Deep vein thrombosis in mice is regulated by platelet HMGB1 through release of neutrophil-extracellular traps and DNA. Sci Rep (2018) 8(1):2068. doi: 10.1038/s41598-018-20479-x 29391442 PMC5794752

[143] Wolach O Sellar RS Martinod K Cherpokova D McConkey M Chappell RJ. Increased neutrophil extracellular trap formation promotes thrombosis in myeloproliferative neoplasms. Sci Transl Med (2018) 10(436):eaan8292. doi: 10.1126/scitranslmed.aan8292 29643232 PMC6442466

[144] Várady CBS Oliveira AC Monteiro RQ Gomes T. Recombinant human DNase I for the treatment of cancer-associated thrombosis: a pre-clinical study. Thromb Res (2021) 203:131–7. doi: 10.1016/j.thromres.2021.04.028 34015562

[145] Graf C Wilgenbus P Pagel S Pott J Marini F Reyda S. Myeloid cell-synthesized coagulation factor X dampens antitumor immunity. Sci Immunol (2019) 4(39):eaaw8405. doi: 10.1126/sciimmunol.aaw8405 31541031 PMC6830514

[146] Kubala MH Punj V Placencio-Hickok VR Fang H Fernandez GE Sposto R. Plasminogen activator inhibitor-1 promotes the recruitment and polarization of macrophages in cancer. Cell Rep (2018) 25(8):2177–91.e7. doi: 10.1016/j.celrep.2018.10.082 30463014 PMC6876299

[147] Yang Y Stang A Schweickert PG Lanman NA Paul EN Monia BP. Thrombin signaling promotes pancreatic adenocarcinoma through PAR-1-Dependent immune evasion. Cancer Res (2019) 79(13):3417–30. doi: 10.1158/0008-5472.CAN-18-3206 PMC6699516 31048498

[148] Schweickert PG Yang Y White EE Cresswell GM Elzey BD Ratliff TL. Thrombin-PAR1 signaling in pancreatic cancer promotes an immunosuppressive microenvironment. J Thromb Haemost. (2021) 19(1):161–72. doi: 10.1111/jth.15115 PMC7790967 33064371

[149] Peng Q Nowocin A Ratnasothy K Smith RA Smyth LA Lechler RI. Inhibition of thrombin on endothelium enhances recruitment of regulatory T cells during IRI and when combined with adoptive treg transfer, significantly protects against acute tissue injury and prolongs allograft survival. Front Immunol (2023) 13. doi: 10.3389/fimmu.2022.980462 PMC9924086 36793549

[150] Li T He S. Induction of IL-6 release from human T cells by PAR-1 and PAR-2 agonists. Immunol Cell Biol (2006) 84(5):461–6. doi: 10.1111/j.1440-1711.2006.01456.x 16869943

[151] Friebel J Wegner M Lammel S Bloebaum L Jakobs K Puccini M. Thrombin receptor protease-activated receptor 1 (PAR1) related cytotoxic CD8+ T cell activity is associated with atrial myopathy and pro-inflammatory immune response in early atrial fibrillation. Eur Heart J (2022) 43(Supplement_2):ehac544.3037. doi: 10.1093/eurheartj/ehac544.3037

[152] Nieman MT. Protease-activated receptors in hemostasis. Blood. (2016) 128(2):169–77. doi: 10.1182/blood-2015-11-636472 PMC4946198 27127302

[153] Adams GN Sharma BK Rosenfeldt L Frederick M Flick MJ Witte DP. Protease-activated receptor-1 impedes prostate and intestinal tumor progression in mice. J Thromb Haemost. (2018) 16(11):2258–69. doi: 10.1111/jth.14277 PMC6214773 30152921

[154] Cantrell R Rosenfeldt L Sharma BK Gourley B Revenko A Monia B. The role of the Thrombin/PAR axis in modulating CD8+ T cell anti-tumor immunity. J Immunol (2022) 208(1_Supplement):121.12–.12. doi: 10.4049/jimmunol.208.Supp.121.12

[155] Lutz MS Klimovich B Maurer S Heitmann JS Märklin M Zekri L. Platelets subvert antitumor efficacy of T cell-recruiting bispecific antibodies. J ImmunoTherapy Cancer. (2022) 10(2):e003655. doi: 10.1136/jitc-2021-003655 PMC8811601 35110356

[156] Servais L Wéra O Dibato Epoh J Delierneux C Bouznad N Rahmouni S. Platelets contribute to the initiation of colitis-associated cancer by promoting immunosuppression. J Thromb Haemostasis. (2018) 16(4):762–77. doi: 10.1111/jth.13959 29369476

[157] Palumbo JS Talmage KE Massari JV La Jeunesse CM Flick MJ Kombrinck KW. Platelets and fibrin(ogen) increase metastatic potential by impeding natural killer cell-mediated elimination of tumor cells. Blood. (2005) 105(1):178–85. doi: 10.1182/blood-2004-06-2272 15367435

[158] Zingoni A Molfetta R Fionda C Soriani A Paolini R Cippitelli M. NKG2D and its ligands: "One for all, all for one". Front Immunol (2018) 9:476. doi: 10.3389/fimmu.2018.00476 29662484 PMC5890157

[159] Palumbo JS Talmage KE Massari JV La Jeunesse CM Flick MJ Kombrinck KW. Tumor cell-associated tissue factor and circulating hemostatic factors cooperate to increase metastatic potential through natural killer cell-dependent and-independent mechanisms. Blood. (2007) 110(1):133–41. doi: 10.1182/blood-2007-01-065995 PMC1896107 17371949

[160] Shi Q Ji T Tang X Guo W. The role of tumor-platelet interplay and micro tumor thrombi during hematogenous tumor metastasis. Cell Oncol (2023) 46(3):521–532. doi: 10.1007/s13402-023-00773-1 PMC12974681 36652166

[161] Metelli A Wu BX Riesenberg B Guglietta S Huck JD Mills C. Thrombin contributes to cancer immune evasion via proteolysis of platelet-bound GARP to activate LTGF-β. Sci Transl Med (2020) 12(525):eaay4860. doi: 10.1126/scitranslmed.aay4860 31915300 PMC7814995

[162] Mariathasan S Turley SJ Nickles D Castiglioni A Yuen K Wang Y. TGFβ attenuates tumour response to PD-L1 blockade by contributing to exclusion of T cells. Nature. (2018) 554(7693):544–8. doi: 10.1038/nature25501 PMC6028240 29443960

[163] Zimmer N Krebs FK Zimmer S Mitzel-Rink H Kumm EJ Jurk K. Platelet-Derived GARP Induces Peripheral Regulatory T Cells-Potential Impact on T Cell Suppression in Patients with Melanoma-Associated Thrombocytosis Cancers. (2020) 12(12):3653. doi: 10.3390/cancers12123653 33291452 PMC7762193

[164] Rachidi S Metelli A Riesenberg B Wu BX Nelson MH Wallace C. Platelets subvert T cell immunity against cancer via GARP-TGFβ axis. Sci Immunol (2017) 2(11):eaai7911. doi: 10.1126/sciimmunol.aai7911 28763790 PMC5539882

[165] Tyagi T Jain K Yarovinsky TO Chiorazzi M Du J Castro C. Platelet-derived TLT-1 promotes tumor progression by suppressing CD8+ T cells. J Exp Med (2022) 220(1):e20212218. doi: 10.1084/jem.20212218 36305874 PMC9814191

[166] Hinterleitner C Strähle J Malenke E Hinterleitner M Henning M Seehawer M. Platelet PD-L1 reflects collective intratumoral PD-L1 expression and predicts immunotherapy response in non-small cell lung cancer. Nat Commun (2021) 12(1):7005. doi: 10.1038/s41467-021-27303-7 34853305 PMC8636618

[167] Rolfes V Idel C Pries R Plötze-Martin K Habermann J Gemoll T. PD-L1 is expressed on human platelets and is affected by immune checkpoint therapy. Oncotarget. (2018) 9(44):27460–70. doi: 10.18632/oncotarget.25446 PMC6007942 29937998

[168] Cho MS Lee H Gonzalez-Delgado R Li D Sasano T Carlos-Alcalde W. Platelets increase the expression of PD-L1 in ovarian cancer. Cancers (Basel). (2022) 14(10):2498. doi: 10.3390/cancers14102498 35626102 PMC9139585

[169] Zaslavsky AB Adams MP Cao X Maj T Choi JE Stangl-Kremser J. Platelet PD-L1 suppresses anti-cancer immune cell activity in PD-L1 negative tumors. Sci Rep (2020) 10(1):19296. doi: 10.1038/s41598-020-76351-4 33168847 PMC7652857

[170] Li Y Xin G Li S Dong Y Zhu Y Yu X. PD-L1 regulates platelet activation and thrombosis. via Caspase-3/GSDME Pathway. Front Pharmacol (2022) 13:921414. doi: 10.3389/fphar.2022.921414 35784685 PMC9240427

[171] Kargl J Busch SE Yang GH Kim KH Hanke ML Metz HE. Neutrophils dominate the immune cell composition in non-small cell lung cancer. Nat Commun (2017) 8:14381. doi: 10.1038/ncomms14381 28146145 PMC5296654

[172] Coffelt SB Wellenstein MD de Visser KE. Neutrophils in cancer: neutral no more. Nat Rev Cancer. (2016) 16(7):431–46. doi: 10.1038/nrc.2016.52 27282249

[173] Jaillon S Ponzetta A Di Mitri D Santoni A Bonecchi R Mantovani A. Neutrophil diversity and plasticity in tumour progression and therapy. Nat Rev Cancer. (2020) 20(9):485–503. doi: 10.1038/s41568-020-0281-y 32694624

[174] Gentles AJ Newman AM Liu CL Bratman SV Feng W Kim D. The prognostic landscape of genes and infiltrating immune cells across human cancers. Nat Med (2015) 21(8):938–45. doi: 10.1038/nm.3909 PMC4852857 26193342

[175] Fang Q Stehr AM Naschberger E Knopf J Herrmann M Stürzl M. No NETs no TIME: crosstalk between neutrophil extracellular traps and the tumor immune microenvironment. Front Immunol (2022) 13:1075260. doi: 10.3389/fimmu.2022.1075260 36618417 PMC9816414

[176] de Andrea CE Ochoa MC Villalba-Esparza M Teijeira Á Schalper KA Abengozar-Muela M. Heterogenous presence of neutrophil extracellular traps in human solid tumours is partially dependent on IL-8. J Pathol (2021) 255(2):190–201. doi: 10.1002/path.5753 34184758

[177] Kaltenmeier C Yazdani HO Morder K Geller DA Simmons RL Tohme S. Neutrophil extracellular traps promote T cell exhaustion in the tumor microenvironment. Front Immunol (2021) 12:785222. doi: 10.3389/fimmu.2021.785222 34899751 PMC8652262

[178] Vesely MD Zhang T Chen L. Resistance mechanisms to anti-PD cancer immunotherapy. Annu Rev Immunol (2022) 40:45–74. doi: 10.1146/annurev-immunol-070621-030155 35471840

[179] Wang H Zhang H Wang Y Brown ZJ Xia Y Huang Z. Regulatory T-cell and neutrophil extracellular trap interaction contributes to carcinogenesis in non-alcoholic steatohepatitis. J Hepatol (2021) 75(6):1271–83. doi: 10.1016/j.jhep.2021.07.032 PMC12888775 34363921

[180] Zhang Y Chandra V Riquelme Sanchez E Dutta P Quesada PR Rakoski A. Interleukin-17–induced neutrophil extracellular traps mediate resistance to checkpoint blockade in pancreatic cancer. J Exp Med (2020) 217(12):e20190354. doi: 10.1084/jem.20190354 32860704 PMC7953739

[181] Shinde-Jadhav S Mansure JJ Rayes RF Marcq G Ayoub M Skowronski R. Role of neutrophil extracellular traps in radiation resistance of invasive bladder cancer. Nat Commun (2021) 12(1):2776. doi: 10.1038/s41467-021-23086-z 33986291 PMC8119713

[182] Mao C Xu X Ding Y Xu N. Optimization of BCG therapy targeting neutrophil extracellular traps, autophagy, and miRNAs in bladder cancer: implications for personalized medicine. Front Med (2021) 8. doi: 10.3389/fmed.2021.735590 PMC8514698 34660642

[183] Liu K Sun E Lei M Li L Gao J Nian X. BCG-Induced formation of neutrophil extracellular traps play an important role in bladder cancer treatment. Clin Immunol (2019) 201:4–14. doi: 10.1016/j.clim.2019.02.005 30771501

[184] Tillack K Breiden P Martin R Sospedra M. T Lymphocyte priming by neutrophil extracellular traps links innate and adaptive immune responses. J Immunol (2012) 188(7):3150–9. doi: 10.4049/jimmunol.1103414 22351936

[185] Huang Y Yuan J Righi E Kamoun WS Ancukiewicz M Nezivar J. Vascular normalizing doses of antiangiogenic treatment reprogram the immunosuppressive tumor microenvironment and enhance immunotherapy. Proc Natl Acad Sci U S A. (2012) 109(43):17561–6. doi: 10.1073/pnas.1215397109 PMC3491458 23045683

[186] van Beijnum JR Nowak-Sliwinska P Huijbers EJ Thijssen VL Griffioen AW. The great escape; the hallmarks of resistance to antiangiogenic therapy. Pharmacol Rev (2015) 67(2):441–61. doi: 10.1124/pr.114.010215 25769965

[187] Jain RK. Normalization of tumor vasculature: an emerging concept in antiangiogenic therapy. Science. (2005) 307(5706):58–62. doi: 10.1126/science.1104819 15637262

[188] Verduzco D Lloyd M Xu L Ibrahim-Hashim A Balagurunathan Y Gatenby RA. Intermittent hypoxia selects for genotypes and phenotypes that increase survival, invasion, and therapy resistance. PloS One (2015) 10(3):e0120958. doi: 10.1371/journal.pone.0120958 25811878 PMC4374837

[189] Li S Zhang Q Hong Y. Tumor vessel normalization: a window to enhancing cancer immunotherapy. Technol Cancer Res Treat (2020) 19:1533033820980116. doi: 10.1177/1533033820980116 33287656 PMC7727091

[190] Tiwari A Oravecz T Dillon LA Italiano A Audoly L Fridman WH. Towards a consensus definition of immune exclusion in cancer. Front Immunol (2023) 14. doi: 10.3389/fimmu.2023.1084887 PMC10073666 37033994

[191] Duan Q Zhang H Zheng J Zhang L. Turning cold into hot: firing up the tumor microenvironment. Trends Cancer. (2020) 6(7):605–18. doi: 10.1016/j.trecan.2020.02.022 32610070

[192] Zhou S Zhang H. Synergies of targeting angiogenesis and immune checkpoints in cancer: from mechanism to clinical applications. Anticancer Agents Med Chem (2020) 20(7):768–76. doi: 10.2174/1871520620666200207091653 32031076

[193] Zheng X Fang Z Liu X Deng S Zhou P Wang X. Increased vessel perfusion predicts the efficacy of immune checkpoint blockade. J Clin Invest. (2018) 128(5):2104–15. doi: 10.1172/JCI96582 PMC5957454 29664018

[194] Allen E Jabouille A Rivera LB Lodewijckx I Missiaen R Steri V. Combined antiangiogenic and anti-PD-L1 therapy stimulates tumor immunity through HEV formation. Sci Transl Med (2017) 9(385):eaak9679. doi: 10.1126/scitranslmed.aak9679 28404866 PMC5554432

[195] Meder L Schuldt P Thelen M Schmitt A Dietlein F Klein S. Combined VEGF and PD-L1 blockade displays synergistic treatment effects in an autochthonous mouse model of small cell lung cancer. Cancer Res (2018) 78(15):4270–81. doi: 10.1158/0008-5472.CAN-17-2176 29776963

[196] Khan KA Kerbel RS. Improving immunotherapy outcomes with anti-angiogenic treatments and vice versa. Nat Rev Clin Oncol (2018) 15(5):310–24. doi: 10.1038/nrclinonc.2018.9 29434333

[197] Yasuda S Sho M Yamato I Yoshiji H Wakatsuki K Nishiwada S. Simultaneous blockade of programmed death 1 and vascular endothelial growth factor receptor 2 (VEGFR2) induces synergistic anti-tumour effect. vivo. Clin Exp Immunol (2013) 172(3):500–6. doi: 10.1111/cei.12069 PMC3646450 23600839

[198] Rini BI Plimack ER Stus V Gafanov R Hawkins R Nosov D. Pembrolizumab plus axitinib versus sunitinib for advanced renal-cell carcinoma. N Engl J Med (2019) 380(12):1116–27. doi: 10.1056/NEJMoa1816714 30779529

[199] Cheng AL Qin S Ikeda M Galle PR Ducreux M Kim TY. Updated efficacy and safety data from IMbrave150: atezolizumab plus bevacizumab vs. sorafenib for unresectable hepatocellular carcinoma. J Hepatol (2022) 76(4):862–73. doi: 10.1016/j.jhep.2021.11.030 34902530

[200] Makker V Rasco D Vogelzang NJ Brose MS Cohn AL Mier J. Lenvatinib plus pembrolizumab in patients with advanced endometrial cancer: an interim analysis of a multicentre, open-label, single-arm, phase 2 trial. Lancet Oncol (2019) 20(5):711–8. doi: 10.1016/S1470-2045(19)30020-8 PMC11686814 30922731

[201] Socinski MA Jotte RM Cappuzzo F Orlandi F Stroyakovskiy D Nogami N. Atezolizumab for first-line treatment of metastatic nonsquamous NSCLC. N Engl J Med (2018) 378(24):2288–301. doi: 10.1056/NEJMoa1716948 29863955

[202] Hack SP Zhu AX Wang Y. Augmenting anticancer immunity through combined targeting of angiogenic and PD-1/PD-L1 pathways: challenges and opportunities. Front Immunol (2020) 11:598877. doi: 10.3389/fimmu.2020.598877 33250900 PMC7674951

[203] Yang T Xiao H Liu X Wang Z Zhang Q Wei N. Vascular normalization: a new window opened for cancer therapies. Front Oncol (2021) 11:719836. doi: 10.3389/fonc.2021.719836 34476218 PMC8406857

[204] Jain RK. Antiangiogenesis strategies revisited: from starving tumors to alleviating hypoxia. Cancer Cell (2014) 26(5):605–22. doi: 10.1016/j.ccell.2014.10.006 PMC4269830 25517747

[205] Pinter M Jain RK. Targeting the renin-angiotensin system to improve cancer treatment: implications for immunotherapy. Sci Transl Med (2017) 9(410):eaan5616. doi: 10.1126/scitranslmed.aan5616 28978752 PMC5928511

[206] McKay RR Rodriguez GE Lin X Kaymakcalan MD Hamnvik OP Sabbisetti VS. Angiotensin system inhibitors and survival outcomes in patients with metastatic renal cell carcinoma. Clin Cancer Res (2015) 21(11):2471–9. doi: 10.1158/1078-0432.CCR-14-2332 PMC4566854 25724518

[207] Izzedine H Derosa L Le Teuff G Albiges L Escudier B. Hypertension and angiotensin system inhibitors: impact on outcome in sunitinib-treated patients for metastatic renal cell carcinoma. Ann Oncol (2015) 26(6):1128–33. doi: 10.1093/annonc/mdv147 25795198

[208] Osumi H Matsusaka S Wakatsuki T Suenaga M Shinozaki E Mizunuma N. Angiotensin II type-1 receptor blockers enhance the effects of bevacizumab-based chemotherapy in metastatic colorectal cancer patients. Mol Clin Oncol (2015) 3(6):1295–300. doi: 10.3892/mco.2015.630 PMC4665652 26807236

[209] Levin VA Chan J Datta M Yee JL Jain RK. Effect of angiotensin system inhibitors on survival in newly diagnosed glioma patients and recurrent glioblastoma patients receiving chemotherapy and/or bevacizumab. J Neurooncol. (2017) 134(2):325–30. doi: 10.1007/s11060-017-2528-3 28631191

[210] Pinter M Weinmann A Wörns MA Hucke F Bota S Marquardt JU. Use of inhibitors of the renin-angiotensin system is associated with longer survival in patients with hepatocellular carcinoma. United Eur Gastroenterol J (2017) 5(7):987–96. doi: 10.1177/2050640617695698 PMC5676550 29163965

[211] Hakim N Patel R Devoe C Saif MW. Why HALO 301 failed and implications for treatment of pancreatic cancer. Pancreas (Fairfax). (2019) 3(1):e1–4. doi: 10.17140/POJ-3-e010 PMC7003617 32030361

[212] Van Cutsem E Tempero MA Sigal D Oh D-Y Fazio N Macarulla T. Randomized phase III trial of pegvorhyaluronidase Alfa with nab-paclitaxel plus gemcitabine for patients with hyaluronan-high metastatic pancreatic adenocarcinoma. J Clin Oncol (2020) 38(27):3185–94. doi: 10.1200/JCO.20.00590 PMC7499614 32706635

[213] Murphy JE Wo JY Ryan DP Clark JW Jiang W Yeap BY. Total neoadjuvant therapy with FOLFIRINOX in combination with losartan followed by chemoradiotherapy for locally advanced pancreatic cancer: a phase 2 clinical trial. JAMA Oncol (2019) 5(7):1020–7. doi: 10.1001/jamaoncol.2019.0892 PMC6547247 31145418

[214] Boucher Y Posada JM Subudhi S Kumar AS Rosario SR Gu L. Addition of losartan to FOLFIRINOX and chemoradiation reduces immunosuppression-associated genes, tregs, and FOXP3+ cancer cells in locally advanced pancreatic cancer. Clin Cancer Res (2023) 2023:OF1–OF15. doi: 10.1158/1078-0432.22633054 PMC10106451 36749873

[215] Magnus N Meehan B Garnier D Hashemi M Montermini L Lee TH. The contribution of tumor and host tissue factor expression to oncogene-driven gliomagenesis. Biochem Biophys Res Commun (2014) 454(2):262–8. doi: 10.1016/j.bbrc.2014.10.041 25450387

[216] Gessler F Voss V Dützmann S Seifert V Gerlach R Kögel D. Inhibition of tissue factor/protease-activated receptor-2 signaling limits proliferation, migration and invasion of malignant glioma cells. Neuroscience. (2010) 165(4):1312–22. doi: 10.1016/j.neuroscience.2009.11.049 19958818

[217] Versteeg HH Schaffner F Kerver M Petersen HH Ahamed J Felding-Habermann B. Inhibition of tissue factor signaling suppresses tumor growth. Blood. (2008) 111(1):190–9. doi: 10.1182/blood-2007-07-101048 PMC2200804 17901245

[218] Alexander ET Minton AR Hayes CS Goss A Van Ryn J Gilmour SK. Thrombin inhibition and cyclophosphamide synergistically block tumor progression and metastasis. Cancer Biol Ther (2015) 16(12):1802–11. doi: 10.1080/15384047.2015.1078025 PMC4847815 26383051

[219] Gong J Drobni ZD Alvi RM Murphy SP Sullivan RJ Hartmann SE. Immune checkpoint inhibitors for cancer and venous thromboembolic events. Eur J Cancer. (2021) 158:99–110. doi: 10.1016/j.ejca.2021.09.010 34662835 PMC9010482

[220] Ye X Hu F Zhai Y Qin Y Xu J Guo X. Hematological toxicities in immune checkpoint inhibitors: a pharmacovigilance study from 2014 to 2019. Hematol Oncol (2020) 38(4):565–75. doi: 10.1002/hon.2743 32383782

[221] Nichetti F Ligorio F Zattarin E Signorelli D Prelaj A Proto C. Is there an interplay between immune checkpoint inhibitors, thromboprophylactic treatments and thromboembolic events? mechanisms and impact in non-small cell lung cancer patients. Cancers (Basel). (2019) 12(1):67. doi: 10.3390/cancers12010067 31881699 PMC7016680

[222] Johannet P Sawyers A Gulati N Donnelly D Kozloff S Qian Y. Treatment with therapeutic anticoagulation is not associated with immunotherapy response in advanced cancer patients. J Transl Med (2021) 19(1):47. doi: 10.1186/s12967-021-02712-w 33516263 PMC7847556

[223] Haist M Stege H Pemler S Heinz J Fleischer MI Graf C. Anticoagulation with factor xa inhibitors is associated with improved overall response and progression-free survival in patients with metastatic malignant melanoma receiving immune checkpoint inhibitors-a retrospective, real-world cohort study. Cancers (Basel). (2021) 13(20):5103. doi: 10.3390/cancers13205103 34680252 PMC8534137

[224] Saidak Z Soudet S Lottin M Salle V Sevestre MA Clatot F. A pan-cancer analysis of the human tumor coagulome and its link to the tumor immune microenvironment. Cancer Immunol Immunother. (2021) 70(4):923–33. doi: 10.1007/s00262-020-02739-w PMC10991611 33057845

[225] Galmiche A Rak J Roumenina LT Saidak Z. Coagulome and the tumor microenvironment: an actionable interplay. Trends Cancer. (2022) 8(5):369–83. doi: 10.1016/j.trecan.2021.12.008 35027336

