# Corrigendum: Reprogramming the immunosuppressive tumor microenvironment: exploiting angiogenesis and thrombosis to enhance immunotherapy

**DOI:** 10.3389/fimmu.2023.1252998

**Published:** 2023-07-13

**Authors:** Areez Shafqat, Mohamed H. Omer, Eman Nayaz Ahmed, Ali Mushtaq, Eman Ijaz, Zara Ahmed, Khaled Alkattan, Ahmed Yaqinuddin

**Affiliations:** ^1^ College of Medicine, Alfaisal University, Riyadh, Saudi Arabia; ^2^ School of Medicine, Cardiff University, Cardiff, United Kingdom; ^3^ Department of Internal Medicine, Cleveland Clinic Foundation, Cleveland, OH, United States

**Keywords:** immunotherapy, tumor microenvironment, angiogenesis, thrombosis, vascular normalization, hypoxia, treatment resistance

In the published article, there was an error in **Figures 1** and **2** as published. **Figures 1** and **2** were incorrectly switched, but the captions are correct.

The corrected **Figures 1** and **2** and their captions appear below:

**Figure 1:**
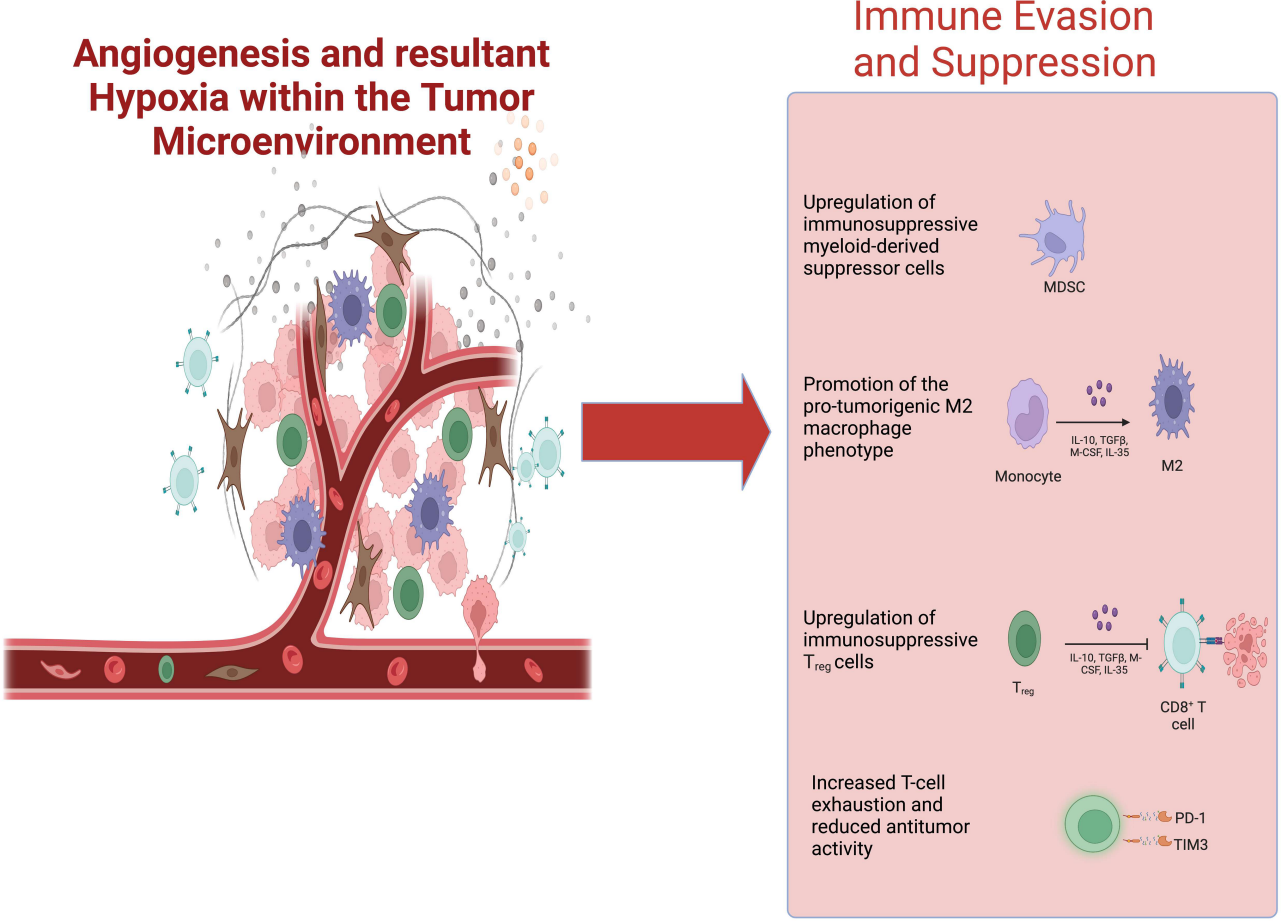
Pro-angiogenic signaling within the TME fosters tumor hypoxia. Tumor hypoxia generates an immunosuppressive tumor microenvironment—promoting the infiltration of MDSCs, M2 TAMs, Tregs, and exhausted CD8+ T-cells—that negatively impacts the efficacy of cancer immunotherapies. TME, tumor microenvironment; MDSCs, myeloid-derived suppressor cells; TAMs, tumor-associated macrophages.

**Figure 2:**
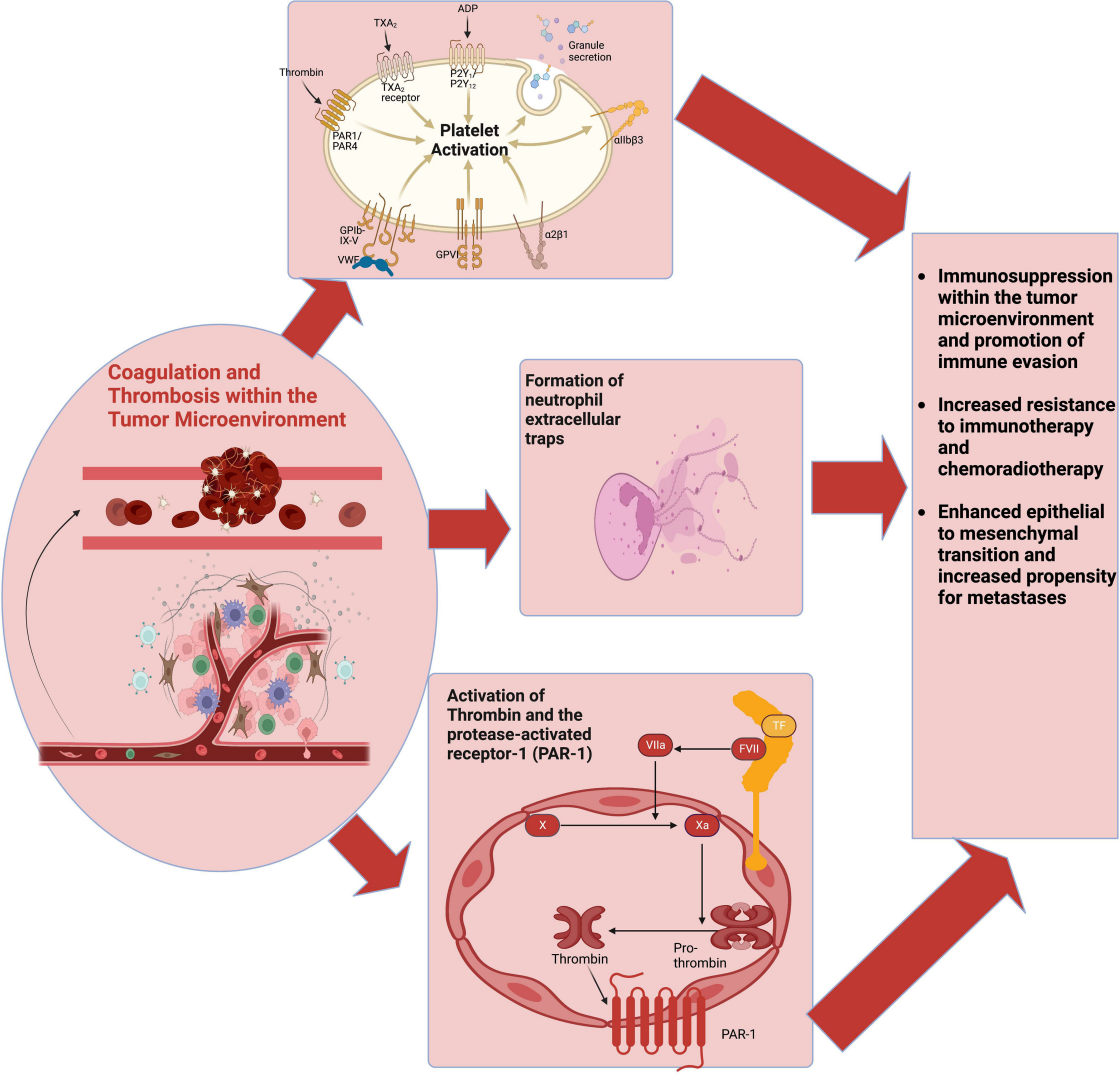
The coagulome describes various components of the tumor microenvironment that modulate coagulation. CAT involves the activation of multiple pathways, including platelet activation, the coagulation cascade, and NETs production. These pathways also interact with various cells of the tumor immune microenvironment to facilitate tumor immune evasion and immunotherapy resistance. CAT, cancer-associated thrombosis; NETs, neutrophil extracellular traps.

The authors apologize for this error and state that this does not change the scientific conclusions of the article in any way. The original article has been updated.

